# Evaluating a therapeutic window for precision medicine by integrating genomic profiles and p53 network dynamics

**DOI:** 10.1038/s42003-022-03872-1

**Published:** 2022-09-07

**Authors:** Minsoo Choi, Sang-Min Park, Kwang-Hyun Cho

**Affiliations:** 1 Department of Bio and Brain Engineering, Korea Advanced Institute of Science and Technology (KAIST), Daejeon, 34141 Republic of Korea; 2 College of Pharmacy, Chungnam National University, Daejeon, 34134 Korea

**Keywords:** Systems analysis, Regulatory networks, Cancer genomics, Cellular signalling networks

## Abstract

The response variation to anti-cancer drugs originates from complex intracellular network dynamics of cancer. Such dynamic networks present challenges to determining optimal drug targets and stratifying cancer patients for precision medicine, although several cancer genome studies provided insights into the molecular characteristics of cancer. Here, we introduce a network dynamics-based approach based on attractor landscape analysis to evaluate the therapeutic window of a drug from cancer signaling networks combined with genomic profiles. This approach allows for effective screening of drug targets to explore potential target combinations for enhancing the therapeutic window of drug responses. We also effectively stratify patients into desired/undesired response groups using critical genomic determinants, which are network-specific origins of variability to drug response, and their dominance relationship. Our methods provide a viable and quantitative framework to connect genotype information to the phenotypes of drug response with regard to network dynamics determining the therapeutic window.

## Abstract (web summary)

A network dynamics-based approach is used to screen for the therapeutic windows of drug combinations and explore the response of the p53 network to drugs in different patients and cell lines.

## Introduction

Cancer is a multifactorial and highly heterogeneous disease with diverse molecular and cellular properties across tumors from different patients and within cancer cells from the same patient^1,2^. This heterogeneity provides not only the basis for personalized precision medicine but also represents a major obstacle for its implementation. The development and clinical application of biomarkers and matched targeted therapies have advanced progress in precision medicine^3^. Advances in molecular profiling and screening methodologies have led to the development and approval of molecularly targeted therapies for clinical use^4,5^. Despite positive responses in some patients, many patients still fail to benefit from these targeted therapies. For example, Herceptin is an approved ErbB-targeted drug. However, only about half of the patients with *ERBB2*-amplified metastatic breast cancer respond to Herceptin^6^. Unfortunately, this lack of response is common for most current targeted therapies. Therefore, improving precision medicine requires a better understanding of the underlying reasons for the variability in drug responses.

The disappointing response rate of targeted therapies is partly due to complex pathway behavior that undergo dynamic crosstalk between in signaling networks, which can bypass the block induced by drug. Such a bypass of the drug’s effects occurs through the multiple feedback loops and alternative pathways that can compensate for therapeutic impact^7–9^. In particular, the hubs of signal transduction networks, such as the transcription factor p53 and the kinases MAPK, and IKK, are critical mediators that are important for cancer development and progression, regulate numerous signaling pathways, and affect distinct phenotypes^10^. Although several cancer genome studies provided insights into genetic basis of cancer heterogeneity^11–13^, they did not address the dynamic, network-specific origin of response variation. Systems analysis of network dynamics using quantitative mathematical modeling is an effective method to evaluate drug perturbation, response variability, and the dynamic changes of complex networks^8,9,14–17^. This approach facilitates comparisons of the impact of different therapeutic strategies. Previously, we showed that functional states of cells and dynamics of biomolecular network within cells can be studied by attractor landscape analysis to identify synergistic drug combinations^18^ and determine drug efficacy and synergism from analysis of the dynamics of cancer cell line-specific networks^19^.

Identifying effective individual drugs and drug combinations is only one part of the process for improving precision medicine. Another important step is determining the optimal dose(s) of the optimal drug(s)^20^. One benefit of precision medicine is more cost-effective patient management by giving the appropriate treatment at the optimal dose to each patient^21^. To accomplish this goal, the balance between drug efficacy and safety must be determined, yet this remains a challenging problem in drug development. Knowledge of the therapeutic window, which is the range of drug doses that treat disease effectively without having toxic effects, is critical to achieving this balance^22^. Despite the importance of the therapeutic window, many studies evaluated the sensitivity of tumor cells to targeted drugs without determining the effect of the drugs on normal cells, which is essential for determining toxicity. This is true for both experimental and mathematical analyses, including our previous works, which predicted drug efficacy in tumor cells without modeling the effect of the drugs on normal cells^18,19^. In addition, many studies do not perform dose–response analysis, which is also needed for determining potency and establishing the therapeutic window.

In this study, we developed a computational framework in which the probabilistic inhibition of a target is considered as an effect of different doses of a drug. With this probabilistic model that incorporates the response to levels of inhibition representing different drug doses, we extended a Boolean simulation and analysis framework^18,19^ to predict three clinically important aspects of drug-mediated target inhibition in a cancer signaling network: efficacy, potency, and toxicity. This approach takes a step toward advancing precision medicine, because it builds on previous drug efficacy studies by incorporating drug potency and dose-related toxicity. We applied this method to the p53 regulatory network, using genomic profiles of cancer patients from The Cancer Genome Atlas (TCGA) and cancer cell lines from Cancer Cell Line Encyclopedia (CCLE)^23,24^ across tumor types. Our extended model and analysis framework, which can evaluate the therapeutic window of drugs on the basis of their effects on the dynamic output of signaling networks, enabled an exploration of potential drug combinations for enhancing the therapeutic window and a new more informative stratification of cancer genomic subtypes according to predicted drug responses. Thus, our approach provides computational simulation results for evaluating therapeutic window of a drug inhibiting the same target across a variety of tumor types to advance progress in precision cancer medicine.

## Results

### Network dynamics-based estimation of therapeutic window

Individualization of dose is a critical for achieving precision medicine and minimizing adverse responses. Generally, there is a population dose that is selected to balance the benefits and risks of adverse effects^25^. However, actual patient responses at this population dose are variable. Some patients will not benefit from the drug at this population dose or any dose. Some patients will develop adverse effects at this dose but respond well to lower doses, and others will have little or no response to the population dose but benefit from higher doses. Therefore, an approach is needed for exploring the diversity of drug responses and predicting the appropriate doses of drugs. In silico virtual experiments that include systemic analysis of dose–response curves is one approach to handling such complex questions. Such an approach would enable rapid and effective examination of different concentrations of drugs and different combinations of drugs at various doses before performing pre-clinical experiments and clinical trials.

**Fig. 1:**
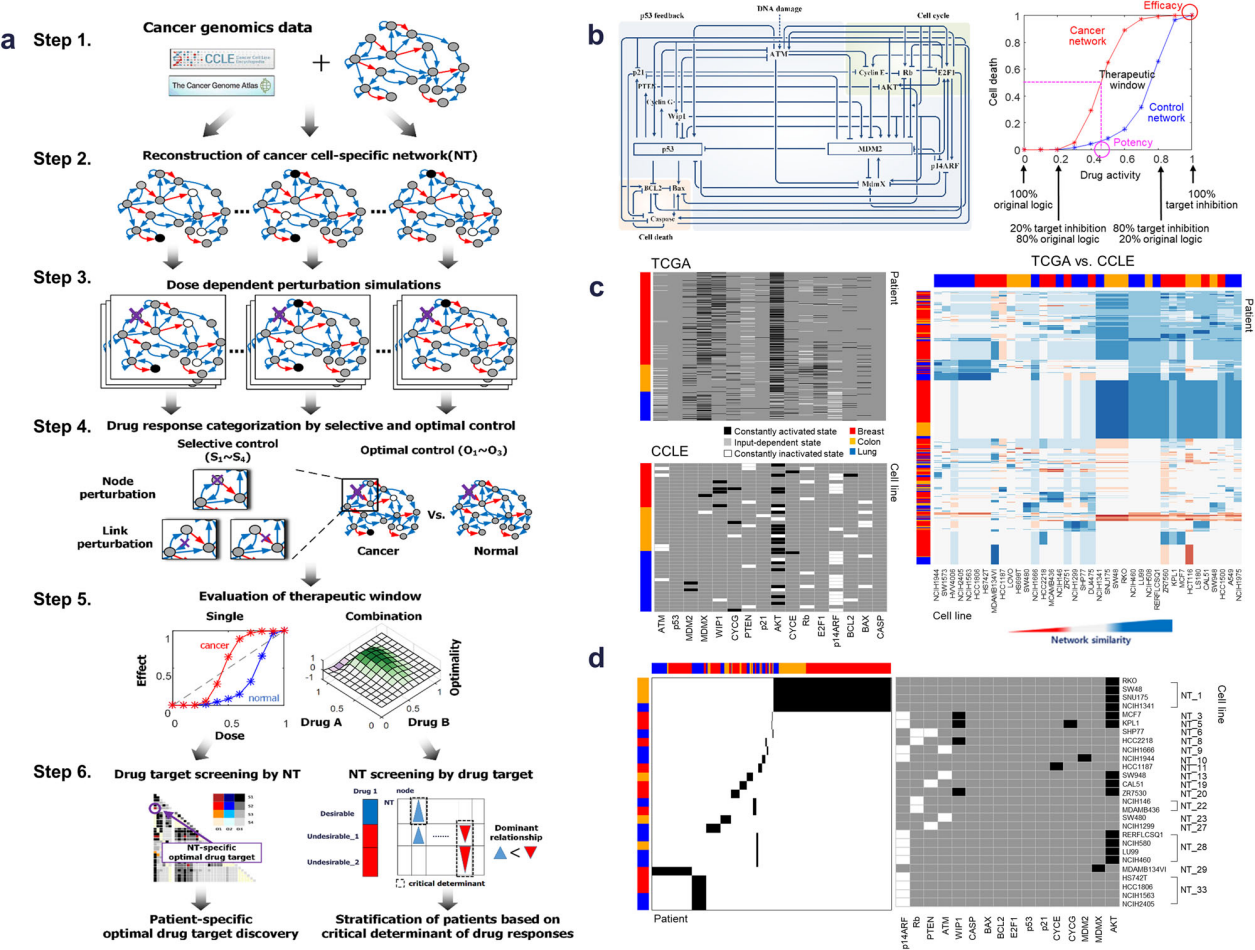
Network dynamics-based estimation of therapeutic window and application to p53 network. **a** Overview of the 6 steps in application of network dynamics-based estimation of therapeutic window for drug-target discovery and stratification of patients. **b** Dose–response curves (right) were obtained from simulations of dose-dependent perturbations in the p53 regulatory network (left). Red and blue graphs denote dose–response curves of cancer and control network, respectively. Efficacy and toxicity were calculated by maximum effects of dose–response curves in cancer and control network, respectively. Potency was calculated by IC50 values of dose–response curves. Therapeutic window was calculated by comparing drug response curves from cancer and control networks. **c** Evaluation cell lines as tumor models by comparison of network features according to genomic alterations from CCLE (cell lines) and TCGA (patient samples). Functional genomic alterations were projected onto the nominal p53 network. Node status of the p53 network was determined based on the genomic data, and assigned in a ternary fashion, such that node activity is either constantly activated (black), constantly inactivated (white), or input-dependent (gray) (left). A systematic comparison of the networks in the tumors and cell lines is performed to identify cancer cell lines with the highest network similarity to those of cancer patients by determining network similarity through correlation index with values from –1 to 1 (from red to blue, right). **d** Selection of cell lines and cancer networks that best match those of patient tumors (left) and genomic alteration profiles (right). Cancer cell lines and patient tumors that have the same node activity profile were matched to an identical single network.

We developed such an approach that extends our network dynamics-based framework^18,19^ by evaluating therapeutic windows for individual drugs and drug combinations. Our approach integrates cancer-specific genomic alterations and is independent of tissue origin and cancer type; instead, cancer cell lines or patients are specifically described by differentially wired networks with distinct network topology that result from the genomic alterations. Our systemic computational approach relies on six steps (Fig. 1a): (Step 1) obtain functional genomic alterations from a cancer genomics database such as CCLE and TCGA; (Step 2) construct cancer cell-specific network models by mapping the functional genomic alterations on the interaction network for each cell line or patient sample; (Step 3) simulate dose-dependent perturbations for each network; (Step 4) score the responses according to two values, one for efficacy (S_1_, S_2_, S_3_, or S_4_) and one for potency and toxicity (O_1_, O_2_, or O_3_) and categorize the responses according to their pairs (S_n_,O_m_); (Step 5) evaluate the therapeutic window for those dose-dependent perturbations predicted from step 4 for each network; and (Step 6) screen patients for optimal drug-target combinations according to their personalized network responses and stratify patients on the basis of dominant effects of genomic alterations on the outcome of inhibition of specific targets (see Methods for a detail description).

### Network dynamics-based analysis of p53 network for estimation of therapeutic window

We sought to apply our approach for analyzing the p53 network (Fig. 1b, left) that we used in our previous studies^18,19^. The p53 network model includes major p53 signaling pathway components, multiple feedback loops and crosstalk between them. In our extended analysis, we analyzed network dynamics in response to dose-dependent perturbation by changing the probability of target inhibition (“OFF” in the network simulations), which is probabilistically implemented as “dose of drug.” For each dose in the range of 0 to 1, we calculated the effect as a ratio of network states that eventually converge to the cell death phenotype (Supplementary Fig. 1 and see Methods). The ratio of 0 indicated that the dose did not converge on a cell death response in any simulation and the ratio of 1 indicated that every simulation of that dose produced a death response. Thus, the effects of target inhibition are described by dose–response curves from which we estimate efficacy (the maximal response) and potency (IC50, the amount of target inhibition that produces a response halfway between baseline and maximum). Based on the computational simulation results, we generated dose–response curves from which we estimated drug efficacy and potency of cancer networks (Fig. 1b, right), and we evaluated the therapeutic window compared to toxicity of control network.

### Evaluating cell lines as tumor models by comparison of network features

Next we performed a systematic comparison of the networks in the tumors and cell lines to identify cancer cell lines with the highest network similarity to those of cancer patients (Fig. 1c). In our previous study^19^, we used genomic data from the CCLE database for human cancer cell lines with wild-type p53 and functional caspases. Then, we constructed cancer cell-specific networks by mapping genomic information from CCLE on the p53 network. In this study, we applied the same approach to generate patient-specific p53 networks using genomic profiles of TCGA patients with breast, colon, or lung cancer. Functional genomic alterations of cell lines and patient tumors for lung, breast, and colon cancers were determined for 56 cell lines (CCLE) and 1,067 patient samples (TCGA) using the method previously described^19^ (Supplementary Data 1). We determined network similarity through correlation index with values from −1 to 1. We found striking differences between cancer cell-line models and most TCGA samples. Our analysis showed that cell lines from the same type of cancer as those in the patients were dissimilar. Furthermore, the differentially wired p53 networks demonstrated intra-cancer type heterogeneity and cross-cancer type similarity. Thus, tissue of origin of the cancer or cell line was not predictive of the properties of the p53 network. We identified 17 distinct cancer-associated p53 networks (Fig. 1d and Supplementary Data 1). For example, network 1 (NT_1) represented three colon cancer cell lines RKO, SW48, and SNU175, one lung cancer cell-line NCIH1341, and multiple breast, colon, and lung cancers from patients. Therefore, we represented the cells and tumors according to their differentially wired p53 network rather than their type of cancer.

### Validation of dose-dependent perturbation simulation

**Fig. 2:**
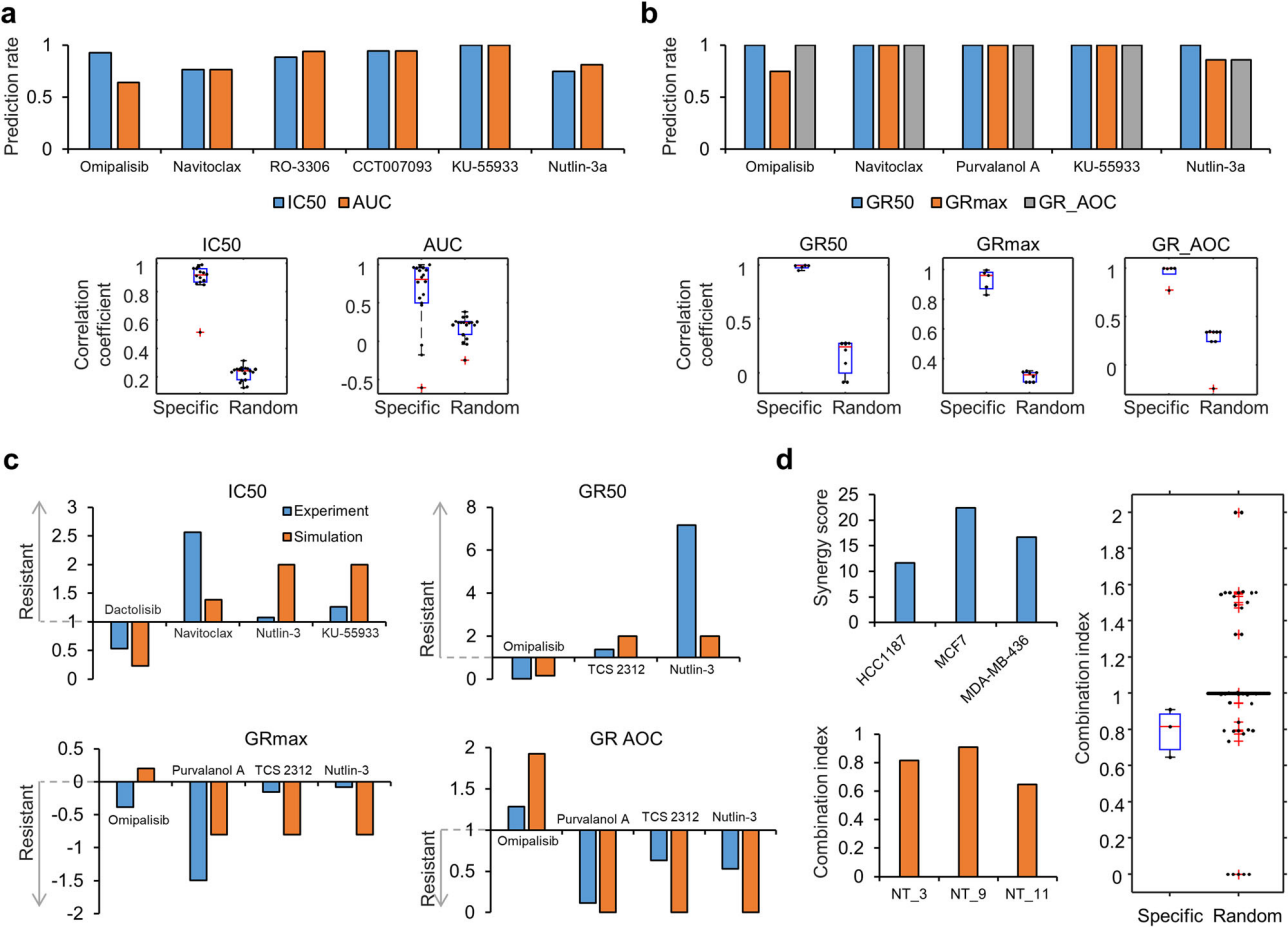
Comparison of perturbation simulation with database for drug response. **a**, **b** Comparison of predicted drug response with (**a**) IC50 and AUC from GDSC data and **b** GR50, GRmax, and GR_AOC from GRbrowser data. Each value from database and simulation of corresponding cancer cell-specific networks was categorized as sensitive or resistant according to a threshold. Prediction rates were calculated for selected drugs (upper panel). Correlation coefficients were calculated between values from database and simulation of corresponding cancer cell-specific networks or 100 networks with random alterations (bottom). **c** Comparison of predicted drug response between simulation results of the control network and experimental data of MCF10A cells. Each sensitivity metric was normalized to its threshold for comparison. **d** Comparison of predicted synergistic effects with DREAM challenge data for combination of AKT_1 and BCL2_2 compounds in HCC1187, MCF7, and MDA-MD-436 cell lines. (left). Synergy score over 0 is categorized as sensitive in DREAM challenge data. Combination index was calculated from simulation of corresponding cell line-specific networks, and combination index under 1 is categorized as sensitive. Combination index from simulation of 100 networks with random alterations was mostly 0, indicating no synergistic effect (right). We used experimental values of the AKT inhibitor omipalisib or dactolisib, the BLC2 inhibitor navitoclax, the CYCE inhibitor RO-3306 or purvalanol A, the MDM2 inhibitor CCT007093, the ATM inhibitor KU-55933 or TCS 2312, and the MDM3-p53 interaction inhibitor nutlin-3 depending on data availability.

We compared the sensitivities of the predicted perturbation responses of the 17 cancer networks with the known drug response sensitivities of the cell lines (Supplementary Data 2). The IC50 and area under the curve (AUC) were obtained from Genomics of Drug Sensitivity in Cancer (GDSC)^26^, and normalized growth rate inhibition (GR) metrics^27^ were obtained from the GRbrowser database^28^. These experimental values and the matched simulated responses were classified as sensitive or resistant by specific thresholds (see Methods). Comparing them, the cell line-specific predicted sensitivities were in agreement with the drug sensitivities reported in both databases (Fig. 2a, b, upper). We demonstrated that random predictions of simulated responses from random networks, whose genomic alterations are randomly generated, showed significantly weak correlations between the experimentally observed and randomly predicted responses than the cell line-specific predictions (*p* < 0.001, Wilcoxon rank sum test, Fig. 2a, b, bottom).

We next validated simulated toxicity using the sensitivities of the predicted perturbation responses of a control network without any genomic alterations (Supplementary Data 2). We assumed the network of MCF-10A cells, a nontumorigenic breast epithelial cell line, as the control network. We used experimental IC50 from the drug response study of MCF-10A^29^, and GR metrics for MCF-10A from GRbrowser database^28^. In most cases, our predictions of the control network were consistent with experimental drug sensitivities (Fig. 2c).

To examine that our approach can be translated to real clinical settings beyond a cellular level, we further validated the simulated toxicity with clinical as well as pre-clinical studies (see Supplementary Note). Considering the maximum tolerated dose (MTD), which is the toxicity measurement for patients in clinical phase I^30^, we calculated simulated MTD (sMTD) of the control network (see Methods). We found that MTD of a BCL2 inhibitor is higher than that of an AKT inhibitor, as sMTD of BCL2 perturbation is higher than that of AKT perturbation in the control network. Toxicity measured in animal studies can contribute to prediction for toxicity in clinical study. The comparison with pre-clinical data using mouse models showed positive correlations between the sMTD and experimental dose for several drugs. Taken together, our simulation approach can provide important information for estimating toxicity in actual clinical tests.

To verify the combination effect of simulated perturbations, we used experimental synergy scores from a DREAM challenge^31^, values of which >0 were classified as synergistic combinations. We obtained the synergy scores data for the inhibitors of AKT and BCL2 in three cell lines that matched to our 17 distinct cancer networks (Fig. 2d, upper left). To compare with combined perturbation of AKT and BCL2 in the cancer networks, we calculated the combination index (CI)^32^, values of which <1 are synergistic combinations (Fig. 2d, lower left). The both resulting CI and synergy scores showed synergism. In contrast, we observed that CIs calculated from random networks were enriched in no synergy (CI = 1) or antagonism (CI > 1) (*p* < 0.001, Wilcoxon rank sum test, Fig. 2d, right).

### Drug response categorization by selective control and optimal control

**Fig. 3:**
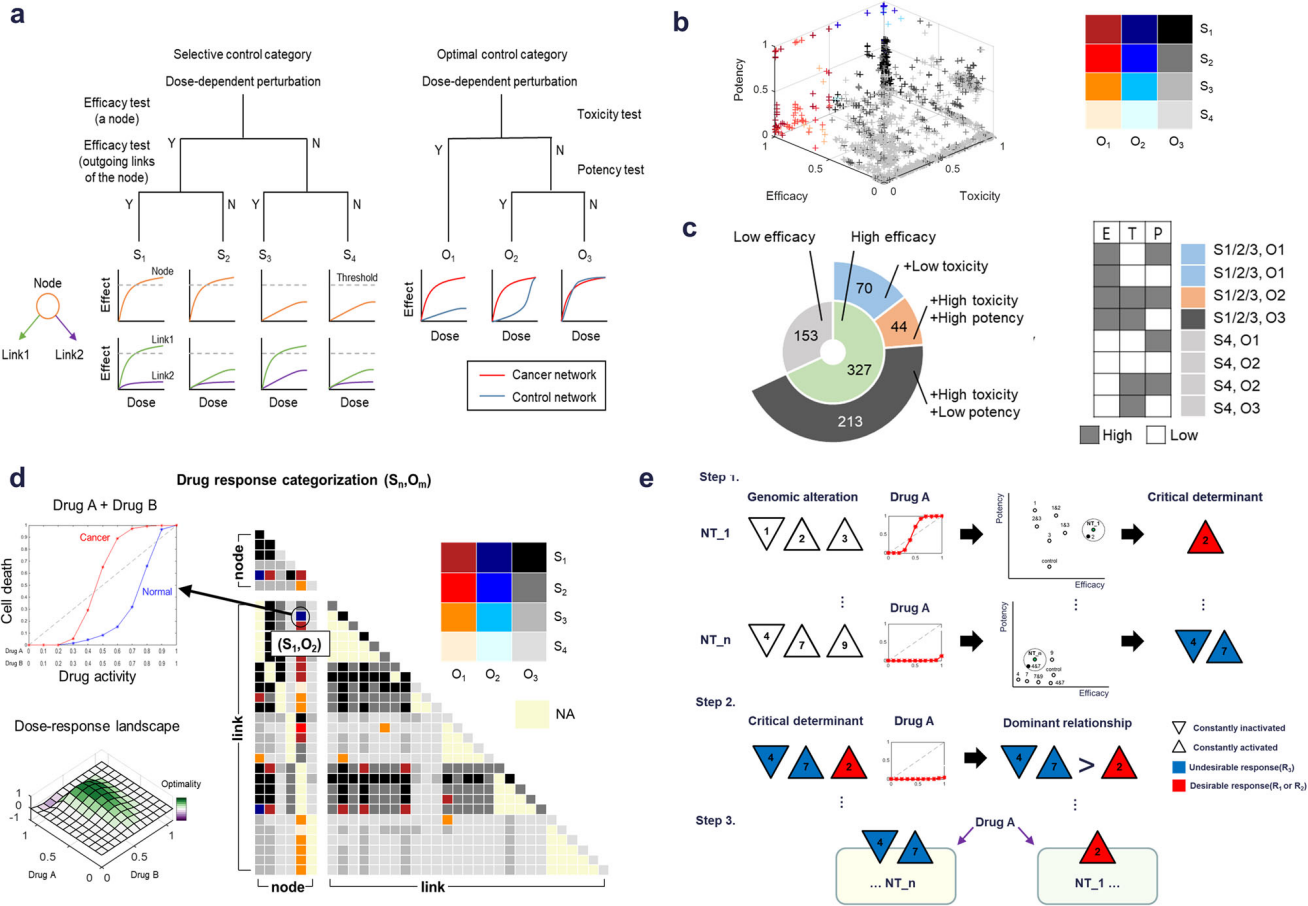
Target screening and patient stratification using drug response categorization. **a** Categorization of for according to “selective control” group as S_1–4_ by efficacy using dose–response curves obtained from simulation of inhibition of a target node and its outgoing links (left). Categorization of responses according to “optimal control” group as O_1–3_ by toxicity and potency by comparing to dose–response curves obtained from simulation of cancer and control networks with inhibition of a target node or link (right). **b** All the drug responses from 17 cancer networks with 480 perturbations were plotted based on efficacy, potency, and toxicity. Colors denote 12 drug response categories (S_n_,O_m_). **c** Distribution of the 480 perturbations in cancer networks with regard to the response categorization. **d** The example triangle map representing all the response categories of 480 drug perturbations to screen for optimal targets for a single cancer-cell network. Each square on the triangle map represents the (S_n_,O_m_) classification obtained from the dose-dependent simulation for corresponding drug or drugs. The NA denotes the case of a link combination in which the two links were from the same node or the case of a node-link mixed combination in which the link from a node were combined with that node. The graph shown in the upper left represents the dose–response curve for corresponding drug or drugs. The graph in the lower left represents the dose–response landscape for corresponding drug combinations. **e** Illustrative workflow to identify critical determinants and their dominance relationship for patient stratification. For each cancer network, we analyze dose–response curves of all the subnetworks, including the control network, in the two-dimensional efficacy–potency plot (step 1). The critical determinant is the common and minimum genomic alteration(s) among the networks exhibit the same drug response with the original cancer network. By determining the effect of combinations of the genomic alterations to the drug response curve, we establish the dominance relationships among them (step 2). We can predict the response of cancer networks and stratify them using critical determinants and their dominance relationship (step 3).

To screening right drug targets for a cancer network, we categorized the simulated perturbation responses on the basis of efficacy, potency, and toxicity (see Methods). We classified the efficacy of the responses into four selective control groups, S_1_–S_4_ (Fig. 3a, left). S_1_ represents a group with an effective response produced by inhibition of the node and the link; S_2_ represents a group with an effective response only produced by inhibition of the node; S_3_ represents a group with an effective response only produced by inhibition of the link; and S_4_ represents a group without any effective response produced by inhibition of the node or the link. Perturbations that produce responses in the S_4_ category have no efficacy by inhibiting the node or its links. In the case of combination drugs, we classified the drug responses by examining whether above conditions were satisfied by at least one drug of combination drugs. Next, we classified the toxicity and potency of the responses into three optimal control groups, O_1_–O_3_ (Fig. 3a, right). O_1_ represents a group with low toxicity at all doses predicted from the control network compared to the cancer network; this group has an optimal drug response with a predicted wide therapeutic window. O_2_ represents a group with a narrower therapeutic window than the O_1_ group due to toxicity predicted with high drug doses in the control network but sensitivity predicted with lose drug doses in the cancer network. O_3_ is an undesirable response group with no therapeutic window; all doses are predicted to produce a toxic effect from the control network. From this pair of groups, a particular perturbation response was categorized into one of 12 response categories based on the selective control group and the optimal control from (S_1_,O_1_) to (S_4_,O_3_).

We categorized all the simulated responses of inhibiting 6 nodes, 27 links from the nodes, and their combinations, representing a total of 480 perturbations for each 17 distinct cancer networks (see Methods; Supplementary Data 3). The simulated potency, efficacy, and toxicity values were plotted in the 3-dimentional space (Supplementary Fig. 2). The results showed that the responses to the various perturbations covered most of the possible space (Fig. 3b). Most of simulated drugs lack a therapeutic window while very few were predicted to have high efficacy, high potency, and low toxicity. Among the 480 perturbations, 153 lacked efficacy (S_4_) in any cancer networks (Fig. 3c). Of the 327 perturbations that had efficacy (S_1_–S_3_) in at least one cancer network, only 70 perturbations also had low toxicity (O_1_). Of the perturbations predicted to have toxicity, 44 perturbations were sufficiently potent to have a potentially safe therapeutic window (O_2_). We defined (S_1_–S_3_,O_1_–O_2_) as desirable (D) responses and the others as undesirable (U) responses.

By representing the (S_n_,O_m_) values for the 480 perturbations for a network on a triangle map, we can visualize and identify the optimal drugs target or combination of targets (Fig. 3d, right). In the triangle map, diagonal line represents the response categories of single target, while the lower triangle area represents those of target combinations: the upper left, lower right, and lower left regions representing node combinations, link combinations, and node-link combinations, respectively. Combination results can be simply analyzed in a dose–response curve (Fig. 3d, upper left) or analyzed in a dose–response landscape, which represents responses of a cancer network over those of the control network at each dose for drugs (Fig. 3d, lower left and Supplementary Fig. 3).

To screening right patients for a drug, we defined the critical determinants of a network to a drug as the minimum genomic alterations that dominantly determined the drug response of the network in terms of efficacy, potency, and toxicity (Fig. 3e, step 1), while our previous study only evaluated the efficacy of drug response^19^. For each cancer network, we generated the subnetworks consisting of all possible combinations of the alterations in each cancer network. After the simulations with a drug perturbation, the dose–response curves from all the subnetworks, including the control network, were analyzed in the efficacy–potency plot (Fig. 3e, step 1, middle). We considered that some networks near the original cancer network in the efficacy–potency plot have the same drug response as the cancer network. The critical determinant was identified as the minimum and common genomic alteration(s) in these neighbor networks, which located within a certain distance from the original cancer network (Fig. 3e, step 1, right and Supplementary Fig. 4). After obtaining all the critical determinants for cancer networks by repeating this procedure, dominance relationship between critical determinants were tested by simulating the virtual network that includes both critical determinants (Fig. 3e, step 2). Using the critical determinants and their dominance relationship, we can predict the response of cancer networks and stratify them (Fig. 3e, step 3).

### Drug-target screening by networks for identifying the right targets

**Fig. 4:**
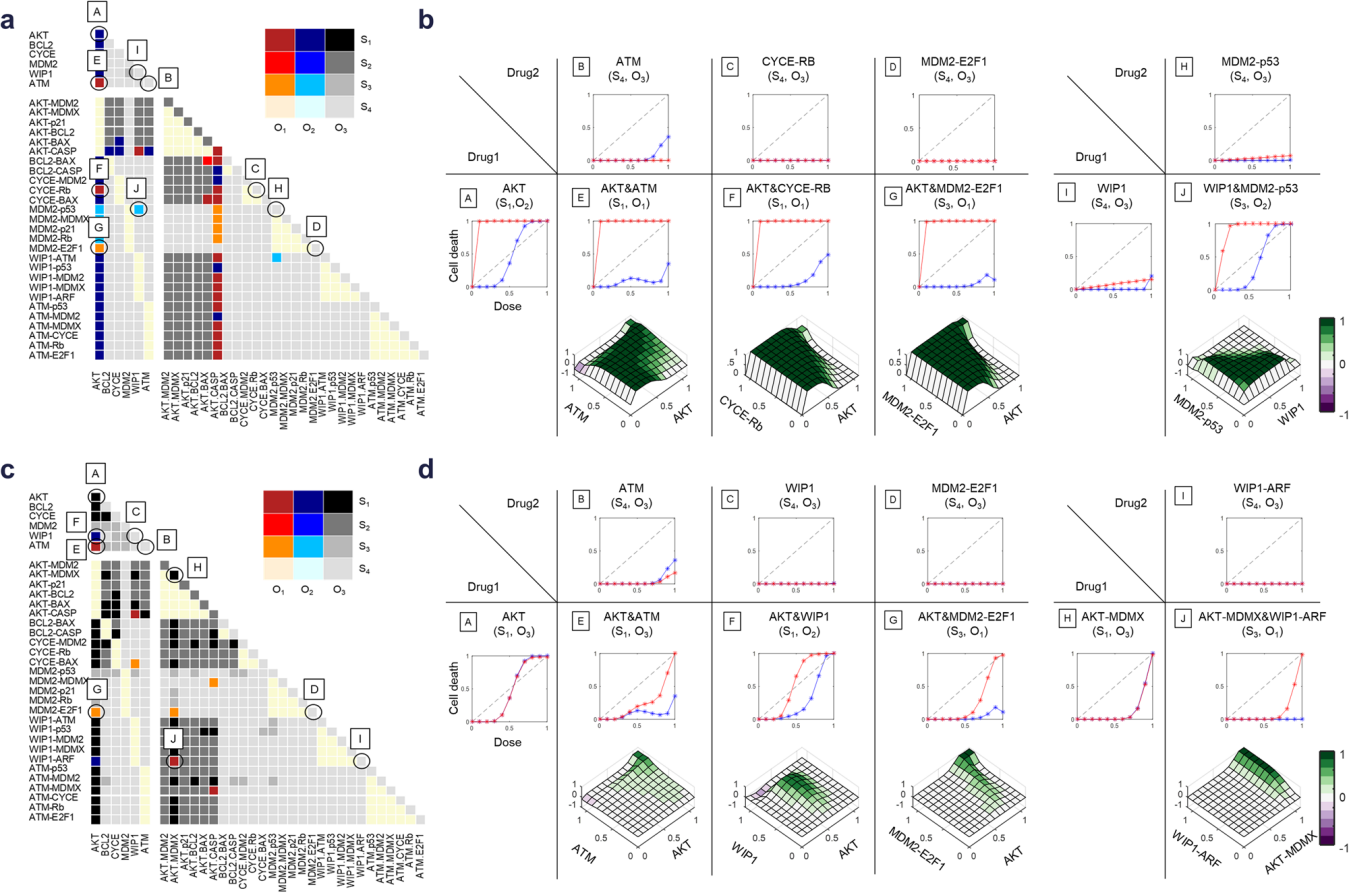
Drug-target screening by cancer cell network responses to identify targets with therapeutic windows. Analysis of the 480 single and combination perturbations in NT_8 (A, B) and NT_9 (C, D). **a**, **c** Triangle maps of the (S_n_,O_m_) classification for NT_8 and NT_9. Capital letters in a and c correspond to graphs in **b**, **d**, respectively. **b**, **d** Dose–response curves and dose–response landscapes for the selected single (A, B, C, D, H, I) and combination (E, F, G, J) perturbations. Red and blue graphs denote dose–response curves of cancer and control network, respectively.

We constructed triangle maps for the (S_n_,O_m_) classifications of the perturbation responses for each cancer network (Supplementary Data 4). From these maps, we could rapidly eliminate the test space by identifying the responses predicted to be clinically viable and also optimal drug-target combinations with improved therapeutic windows. As an example, we highlight responses in NT_8 and NT_9 (Fig. 4).

For perturbations of single nodes in NT_8, only AKT perturbation showed a D response (O_2_) while all others lacked efficacy (S_4_) (Fig. 4a). Evaluation of the dose–response curve revealed that AKT perturbation has a high potency and efficacy but also exhibits toxicity in the control network (Fig. 4b). From the triangle map, the combinations of AKT perturbation with inhibitions of ATM, CYCE-RB link, and MDM2-E2F1 link were identified as producing O_1_ response (Fig. 4b, A). The single perturbations of these three targets showed no efficacy (Fig. 4b, B–D). However, combination of one of them with AKT perturbation reduced the toxicity, resulting in an enhanced therapeutic window compared with single inhibition of AKT (Fig. 4b, E–G). We also identified an effective combination arising from a pair of ineffective single perturbations is exemplified by node inhibition of WIP1 (Fig. 4b, I) and link inhibition of MDM2-p53 (Fig. 4b, H), which individually showed no efficacy (S_4_,O_3_). Their combination showed a D response (O_2_), having high efficacy and potency with a therapeutic window (Fig. 4b, J).

In contrast to the (S_1_,O_2_) classification of AKT perturbation in NT_8, this perturbation was classified as (S_1_,O_3_) in NT_9 (Fig. 4c) that shows high efficacy but no higher potency compared to the control network and thus no therapeutic window (Fig. 4d, A). However, several combinations with AKT perturbation showed D responses compared to single perturbations (Fig. 4c). Combined perturbations with inhibition of ATM (Fig. 4d, E) or MDM2-E2F1 (Fig. 4d, G) resulted in an O_1_ response with low toxicity, like as in NT_8. Combined perturbations with inhibition of WIP1 (Fig. 4d, C) resulted in an O_2_ response by higher potency than the control network.

In NT_9, combined link inhibition of WIP1-ARF and AKT-MDMX resulted in an optimal O_1_ response (Fig. 4d, J). Individually, inhibition of these links was each in the undesirable O_3_ category: Single WIP1-ARF perturbation was classified as (S_4_,O_3_), because it lacked efficacy and toxicity, and single AKT-MDMX perturbation was classified as (S_1_,O_3_), because it has high efficacy and high toxicity (Fig. 4d, H and I). However, combined perturbation of them showed high efficacy without toxicity (S_3_,O_1_) (Fig. 4d, J).

The above selected examples demonstrated that our framework can reveal various strategies for enhancing the therapeutic window of a response by combining drugs. In addition, the dose–response landscapes showed distinct dosing patterns; some are broad and some are very narrow. A comparison of the dose–response landscapes for ATM or MDM2-E2F1 combination with AKT in NT_8 and NT_9 revealed how different cancer networks exhibit different dose sensitivities even to treatments that are optimal for both networks. The two combinations with AKT inhibition showed broadly effective doses for each of the two targets in NT_8 (Fig. 4b, E and G) in contrast to the therapeutic windows exhibited narrow ranges of doses in NT_9 (Fig. 4d, E and G). In addition, the optimal doses for combinations are also different: In NT_9, the combination of AKT-MDMX and WIP-ARF inhibition required a high dose of AKT-MDMX inhibition but only a low dose of WIP-ARF inhibition was needed (Fig. 4d, J).

### Identification of critical determinant for defining the right patient

**Fig. 5:**
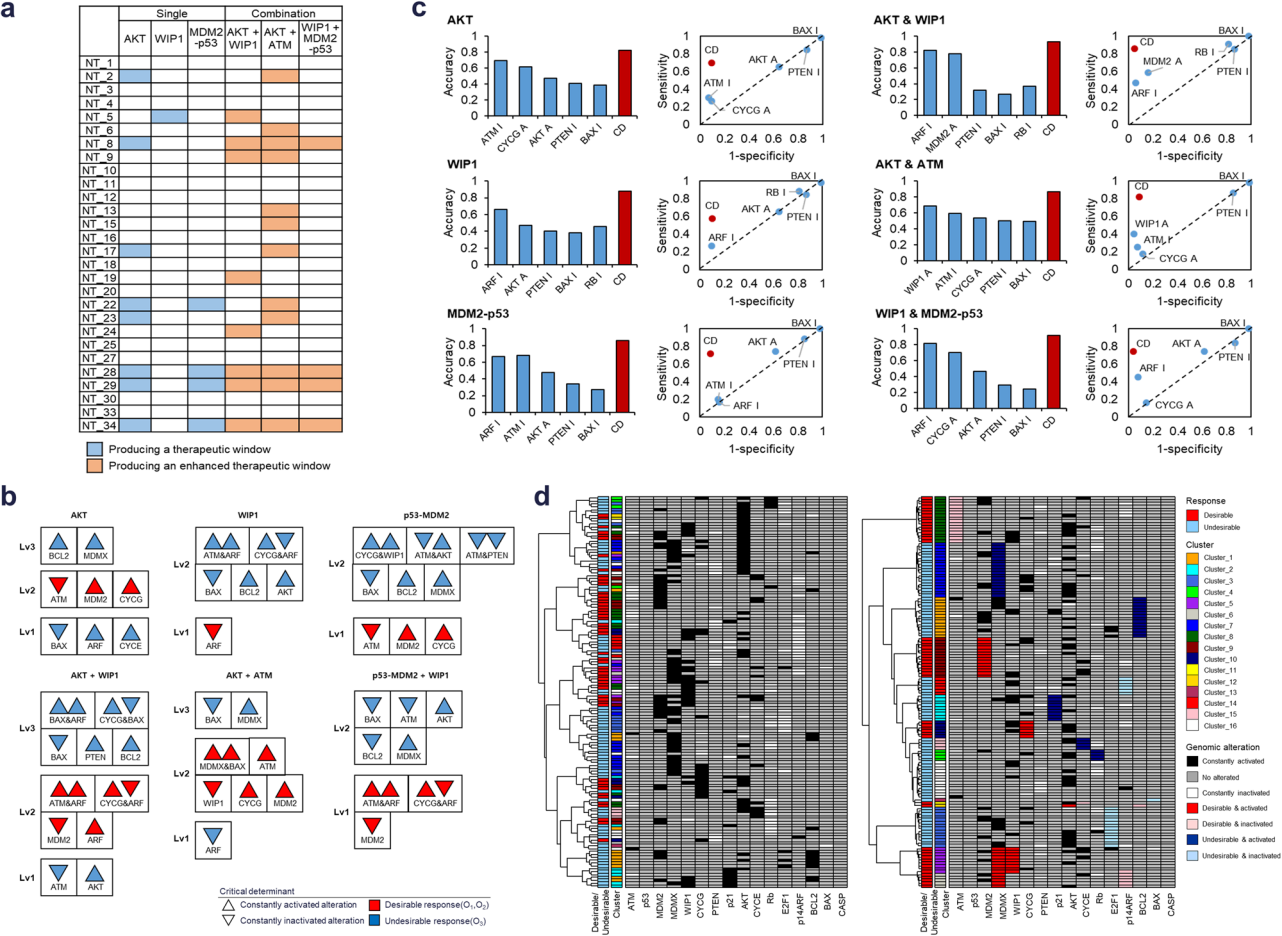
Prediction of drug response and stratification of patients based on critical determinants. **a** The selected 3 single and 3 combination perturbations from the cell line-specific networks. **b** Critical determinants and their dominance relationship obtained from the cell line-specific networks. Upward pointing triangle indicates an activating genetic alteration; downward pointing indicates inactivating genetic alteration. Red indicates D response; blue indicates U response. Critical determinants at higher levels (Lv) are dominant over those at lower levels in determining the drug response. **c** Prediction of drug responses in the patient-specific networks using statistically identified markers or the dominance relationship between critical determinants (CD). **d** Stratification of patient-specific networks to the response of AKT inhibition based on unbiased genetic alterations (left) or on the dominance relationship between critical determinants obtained from patient-specific networks (right). The first column shows D or U responses of patient-specific networks. The second column shows group for each critical determinant. The remaining columns show genetic alterations in patient-specific networks (black: constantly activated alteration, white: constantly inactivated alteration, gray: no alteration). Among the genomic alterations, critical determinants for each patient are highlighted in colors (light blue: constantly inactivated alteration as an U response marker, dark blue: constantly activated alteration as U response marker, red: constantly activated alteration as D response marker, pink: constantly inactivated alteration as D response marker). The cluster 1–15 are notated by the critical determinants in Supplementary Fig. 5 while the cluster 16 has no critical determinant.

We investigated whether critical determinants obtained from the cell line-specific networks can stratify the patient-specific networks by predicting D or U responses. We selected AKT and WIP1 as single-node perturbations and p53-MDM2 as single-link perturbation that showed D responses with therapeutic windows in a subset of the cell line-specific networks (Fig. 5a). Among the possible combinations of the above three single perturbations, we selected AKT + WIP1, AKT + ATM, and p53-MDM2 + WIP1 perturbations that showed enhanced therapeutic windows compared to each of these as single perturbations in a subset of the cell line-specific networks (Fig. 5a).

As illustrated in Fig. 3d, we obtained the critical determinants of the cell line-specific networks for the selected three single and three combination perturbations, and identified the dominance relationship between them (Fig. 5b). For instance, in the case of AKT perturbation, a network with activating alterations of BLC2 or MDMX always showed an U response, because these critical determinants were the most dominant (denoted as Lv3). In the absence of these alterations, a network with an inactivating alteration of ATM or an activating alteration of MDM2 or CYCG showed a D response (denoted as Lv2). A network without critical determinants showed the same response as the control network, which is an U response. Some critical determinants were composed of a combination of genomic alterations such as inactivating alterations of both ATM and ARF in the case of WIP1 perturbation. These results suggested that drug responses are not determined by a single genetic alteration but by multiple genetic alterations and interactions between them.

We hypothesized that predicting drug responses based on the dominance relationship of critical determinants is more accurate than predicting responses based on a single biomarker. Conventional methods of predicting drug responses used statistically enriched genomic markers in a patient group with a similar empirical response to the drug^3^. Using this approach, we identified 5 conventional biomarkers for each perturbation by selecting genomic alterations enriched in either groups of the 28 cell line-specific networks with D or U response (Supplementary Data 5). Next, we compared the performance of predicting whether drug responses of the 137 patient-specific networks are desirable (Supplementary Data 6). For all 6 perturbations, drug response predictions by critical determinants and dominance relationships were more accurate with higher sensitivity and specificity than those predictions made by one of the conventional genomic markers (Fig. 5c), indicating that our approach is more appropriate to be translated clinically for patient stratification according to network-specific origins of heterogenous drug responses.

Accumulated clinical information on patients’ drug responses and their genomic profiles can be used to contribute to the identification of more comprehensive critical determinants and dominance relationship. For instance, we re-identified the critical determinants and their dominance relationships for AKT perturbation in the 137 patient-specific networks, which were expanded with additional determinants and dominance levels compared to Fig. 5b (Supplementary Fig. 5). To demonstrate patient stratification results obtained by our approach, we first stratified the 137 patient-specific networks by hierarchical clustering of their genomic information for comparison (Fig. 5d, left). Although similarly clustered patients shared common genomic alterations, their drug responses to AKT perturbation were inconsistent. We next stratified these patient-specific networks using the re-identified critical determinants and their dominance relationships (Fig. 5d, right). The resulting clusters were perfectly matched with the pattern of D and U responses, indicating an ideal stratification. We found that a common alteration in a large set of patients (e.g., activating alteration of AKT) do not always dominantly determine a drug response. Furthermore, patients with U responses may be resulted from different critical determinants (e.g., activating alteration of E2F1 or MDMX), which can inform that different strategies are required for each patient group to improve the drug responses. These results indicated that patient stratification for drug responses using critical determinants provides more detailed and accurate information than that achieved with stratification by common genomic alterations.

### in silico basket trial based on the network dynamics for optimal therapeutic strategies

To realize precision medicine, we need to identify appropriate targets for intervention, identify drugs with efficacy for those targets, optimize for safety and toxicity, and determine which patients will benefit from the therapy. Our framework identified three different strategies for enhancing the therapeutic window by combination treatments: improving efficacy in the cancer network, increasing potency in the cancer network than the control network, or reducing toxicity in the control network (Supplementary Fig. 6, Supplementary Note, and Supplementary Data 7). Furthermore, we found that selective inhibition of specific links between molecules in a network may provide a path for further therapeutic improvement (Supplementary Fig. 7 and Supplementary Note). Anti-cancer drug of such selective link inhibition is still relatively uncommon, although the p53-MDM2 inhibitor Nutlin-3 represents one example^33^.

**Fig. 6:**
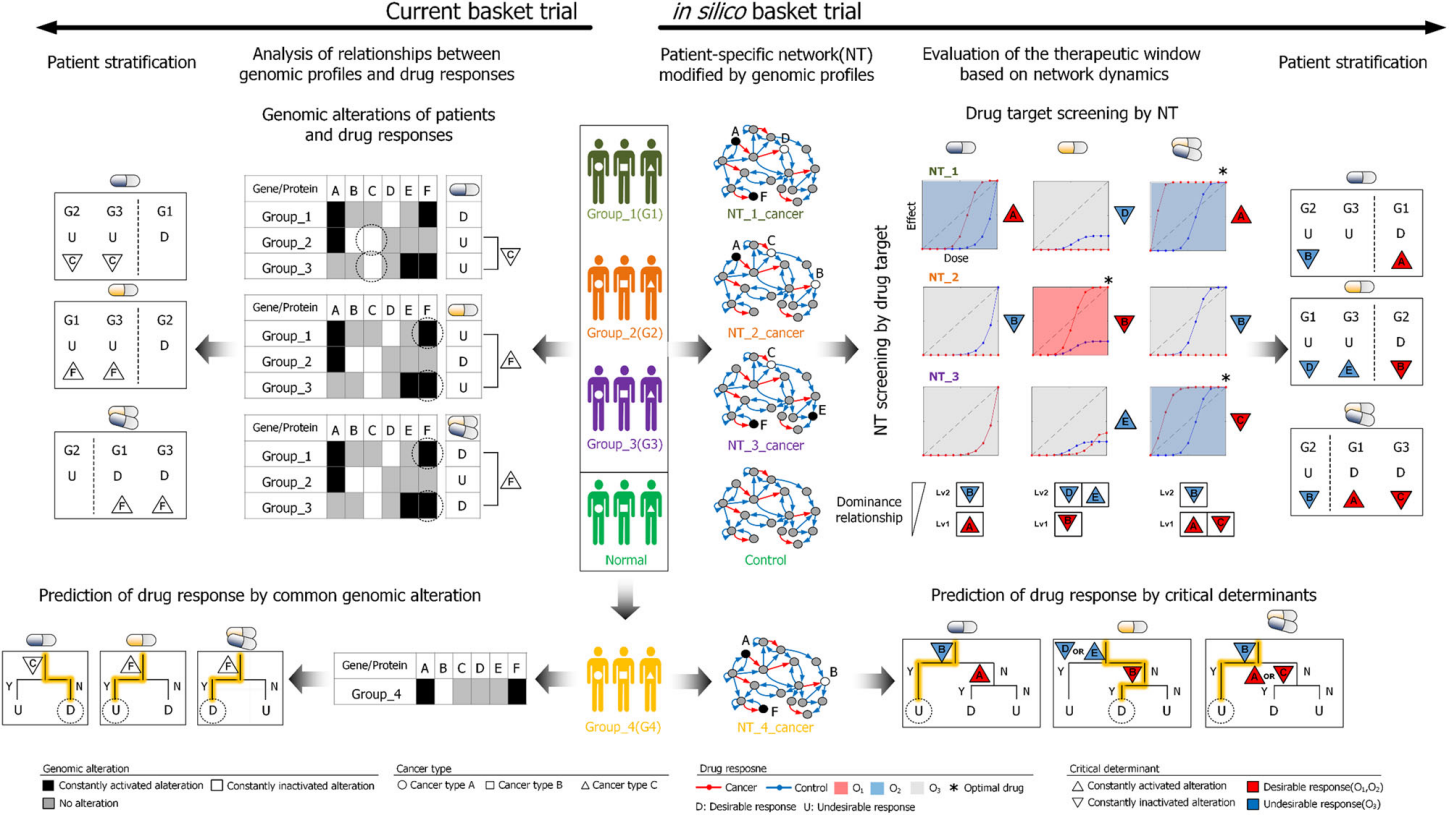
Overview of network dynamics-based evaluation of the therapeutic window and stratification of patients for advancing precision medicine. The patients with different cancer types are clustered into groups, G1, 2, and 3, according to their genomic profiles. The current basket trial analyzes relationships between the patient group’s genomic profile and given drug responses (left). On the other hand, our in silico basket trial analyzes the dynamics of patient group-specific networks, NT_1, 2, and 3 representing G1, 2, and 3, respectively, through virtual experiments (right). The two approaches lead to different drug response predictions for the new group, G4 (bottom).

Compared to the current basket trial, our approach might be applied to in silico basket trial (Fig. 6). The basket trials consider cancer patients with identical genomic profile as one group for a drug treatment regardless of their cancer types. These patient groups are stratified by biomarkers that are statistically enriched in common response groups (Fig. 6, left). For the first blue drug, G2 and G3 exhibit U responses and alteration of C is common between two groups. For the second yellow drug, G1 and G3 exhibit U responses and alteration of F is common between two groups. For the third drug combination, G1 and G3 exhibit D responses and alteration of F is common between two groups. Using theses common biomarkers C and F, the patient groups were classified.

On the other hand, our in silico basket trial considers not only the genomic profile, but also the network dynamics of patients through virtual experiments (Fig. 6, right): generating patient group-specific networks by mapping of genomic profiles; categorizing the simulated response of the networks for candidate drugs; screening optimal drug target for each patient group; screening optimal patient group for candidate drugs. For the first drug, the alteration of A is a critical determinant for the D response of NT_1. NT_2 also includes the alteration of A, but NT_2 exhibits the U response due to the more dominant critical determinant, which is the inactivated alteration of B, suggesting a genetic origin of the drug resistance mechanism. Since NT_3 contains no critical determinant, the response of NT_3 is the same as that of the control network. For the second drug, NT_1 and 3 exhibit the U response due to the alterations of D and E, respectively, whereas NT_2 exhibits the D response due to the alteration of B. In these cases, the alteration of B can be a critical determinant for either U or D responses depending on the drug. For the drug combination, NT_1 and 3 exhibit the D responses for the combination drug due to the alterations of A and C, respectively. Although NT_2 includes both alterations of A and C, NT_2 exhibits the U response for the combination drug due to the more dominant alteration of B. The patient groups were classified using both the critical determinants and their dominance relationships. We note that both first and combination drugs resulted in D responses, but the combination drug treatment is more optimal for NT_1 considering its broader therapeutic window than that of the first drug.

Although two approaches reached the same stratification result, criteria to stratify were different: statistical biomarkers or critical determinants for current or our approaches, respectively. They resulted in different predictions for drug responses of new patient group (Fig. 6, bottom). For this group with alterations of A, B, and F, the biomarkers identified by the current approach predict D, U, and D responses against treatments of the first drug, second drug, and combination of them, respectively; but their response were U, D, and U, respectively, as predicted by the critical determinants and their dominance relationship identified by our approach. This comparison exemplifies that statistically identified single genomic markers are insufficient to find origin of variation in complex network for predicting drug responses. For instance, the biomarker C and F identified by the current approach actually does not affect network dynamics in response to the drugs and their combinations. Taken together, our in silico basket trials can identify optimal drug target with optimal dose for each patient group and can stratify patients in detail according to their network dynamics.

## Discussion

Cancer precision medicine aims to provide the right dose of the right drug for the right patient, based on the genetic profiles of the cancer and the individual^34^. A key contribution of our study is that we developed a framework to estimate therapeutic window from the dynamic response of a signaling network. We applied attractor landscape analysis to implement this framework. In our approach, the therapeutic window is represented as the range of amount for inhibiting the targeted node or link. We analyzed drug response profiles and evaluated the therapeutic window of a drug or two-drug combination based on the dynamics of the patient-specific network generated with the patient’s genomic profile. We then categorized the drug responses according drug response curves, through which we identified drug efficacy, potency, and toxicity. From the therapeutic window, we identified which drug or combination of drugs, along with the optimal dose or doses, is likely to be most effective for an individual patient. We also determined network dynamics-based biomarkers, which are genomic alterations that function as critical determinants of the drug response and used the dominance between these determinants to understand patient variability in drug response. Based on these results, we proposed a method for patient stratification to match the right drug to the appropriate patient group.

In silico modeling of drugs that target nodes can produce full inhibition of the targeted node that blocks all the regulatory functions (out-going links) of the target. However, full inhibition of the target do not always lead to desirable results^35^. For example, full inhibition of a node may have high efficacy and also high toxicity. Many nodes in a network are connected to more than one downstream target and selective inhibition of one of these paths may have a better therapeutic profile. From a network perspective, this selective inhibition may be possible through link inhibition rather than node inhibition. Through the incorporation of selective control analysis, our framework enabled the analysis of the effect of inhibition of nodes or links from the nodes. We showed that our framework enabled the identification of selective link inhibition rather than node inhibition to achieve an enhanced therapeutic window. Another application of our framework is the identification of drug combinations with enhanced therapeutic windows compared to the windows of either drug individually^36^. Combination treatments are particularly attractive, because they tend to be effective at lower doses, which can reduce toxicity and overcome resistance to high-dose single-drug treatment. The purpose of the combination can be broadly divided into two. Here again, our approach revealed that many synergistic combinations involved link inhibition. Thus, selective control analysis can predict synergistic treatment strategies.

An important issue in cancer precision medicine is stratification of patients by grouping tumors into clinically and biologically meaningful subtypes according to the similarities among their genomic or molecular profiles. Different approaches based on various data sources have been proposed. Often, these approaches result in a single statistically significant biomarker for stratification of patients^3^. However, due to the intra- and inter-heterogeneities of tumors, it is challenging to define an entire tumor subtype on a single molecular event. Thus, efforts now search for combinations of genomic driver events or network modules to stratify patients. In this study, we used patient-specific network analysis to define critical determinants of drug response and the hierarchical relationship among them. With these dominance-related critical determinants, we analyzed and explained the diversity of drug responses. Indeed, patients with the same predicted drug response did not correlated with patient groups defined by conventional biomarkers, but these patients correlated with grouped defined by critical determinants.

Although our framework represents a substantial advance in performing in silico clinical trials, our research has several limitations for implementation. First, we determined toxicity by testing a single normal cell line without matching the normal cell to the cell type of the tumor or of other normal types of cells. In fact, toxicity can affect normal cells in the tissues with the tumors or other organs, such as heart, liver and kidney. Our study does not incorporate organ-specific toxicity. Second, we assumed that the genetic network of the normal cell is stable, lacking genetic variation. However, even normal cells have genetic variation^37^. A future improvement would be to use individual variants from each patient’s normal samples to construct distinct normal networks as controls for each person. Third, we evaluated only one cancer-relevant network, the p53 network, and thus may miss genetic variations that influence drug response. Future studies can either analyze different cancer-relevant networks or expand the p53 network to include other relevant networks.

Nevertheless, we expect that our approach will make an important contribution to advancing precision medicine. Our approach reflects the characteristics of cancer cells from the viewpoint of network dynamics and predicts a therapeutic window, which brings the process a step closer to the implementation of precision medicine than the existing methods^3^. As more quantitative genomic data become available for diseases and disease-associated cellular processes, our approach can be adapted for different disease-relevant networks to investigate effects of genomic alterations on response to disease treatment and identify appropriate, patient-specific drug treatment. In addition, given that cancers with common genetic and/or transcriptome profiles exhibited similar drug responses^23,38^, our approach may guide drug repurposing in cancers mapped to the same network based on existing drug information for cancers mapped together or simulated perturbation results in that network.

## Methods

### Boolean network modeling of the p53 network

The downregulation of the *TP53* gene encoding p53 or mutations that impair p53 function are among the most-common and best-characterized genomic alterations associated with cancer^39^. This protein is a tumor suppressor that is a critical hub in cellular processes, such as regulation of the cell cycle, the response to DNA damage, and induction of apoptosis^40^. To investigate the dynamic process of drug responses with respect to the p53 network including multiple feedbacks, we used a p53 network model taken from an updated version of that in our previous studies^18,19^. It is a simplified Boolean network model consisting of 16 nodes with multiple feedback loops through p53 for analyzing the p53 network dynamics (Supplementary Data 8). In our previous studies, we modeled the network dynamics using a deterministic Boolean network with a set of logic equations defined on the basis of biological evidence. In our p53 network model, each node is associated with a logic table that determines the output node for a given input. The basal levels of the nodes and the interaction weights between nodes were determined as the minimal integer values that can represent the logical regulatory relationships in accord with previous experimental observations. Network dynamics were modeled by updating the Boolean functions, triggering system transits from the initial state to the final state, in which a network state is a collective binary representation of all variables. The state of each node can be either ON (1) or OFF (0) at each time step. To compute the network dynamics, we constructed a weighted sum logic with a weight for each link and the basal level of each node. More details on the state transition logic, together with the interaction weights and basal levels, are provided in Supplementary Data 9. In this study, we extended the p53 network perturbation analysis for investigating network dynamics in response to dose-dependent perturbation by changing the effect of target inhibition, which is probabilistically implemented as a dose–response curve.

### Selection of the functional genomic data and mapping to p53 network

DNA copy number, somatic mutation, and mRNA expression data were analyzed for all the patients from TCGA and cell lines from CCLE within 3 distinct tissue origins (breast, colorectal, and lung). The datasets were obtained using cBioPortal (https://www.cbioportal.org/) and the listings are “Colorectal Adenocarcinoma (TCGA, PanCancer Atlas)”, “Lung Adenocarcinoma (TCGA, PanCancer Atlas)”, “Breast Invasive Carcinoma (TCGA, PanCancer Atlas)”, and “Cancer Cell Line Encyclopedia (Novartis/Broad, Nature 2012)”. We selected 1,068 patients from TCGA and 56 cell lines from CCLE that have wild-type p53 and caspases. We consider all available genomic data types in our analysis, including genome-wide DNA copy number information and mutation data for genes associated with the p53 pathway. To focus on mutations most likely to be functional, mutations in introns, untranslated regions, flanking, and intergenic regions, as well as silent and RNA mutations, were excluded. The CCLE database provides the number of reads per base in the sequenced regions, so the number of bases covered was given by the number of positions with one or more reads. To filter out events that were likely non-functional, only genes with copy number alteration (CNA) that have concordant changes in mRNA expression, when compared to wild-type cases, were selected. In total, we curated 191 candidate functional alterations. These alterations were considered in a binary fashion, such that an alteration either occurred or did not occur in a given cancer cell line. The resulting set of functional genomic alterations thus provides a concise genomic description of the cancer cell lines.

Functional genomic alterations were projected onto the p53 network. Node status of the p53 network was determined based on the genomic data, and assigned in a ternary fashion, such that node activity is either constantly activated (A), constantly inactivated (I), or input-dependent (N). Differentially wired p53 networks were constructed by applying this mapping. For patients or cell lines have the same alteration profiles, they are mapped to an identical single network. Through this mapping process, 222 patient networks and 33 cell line networks were uniquely constructed. Attractor landscape analysis for each network was performed to calculate the basin of apoptosis states in which caspase is ON. Networks with the basin of apoptosis larger than half were excluded, resulting in 137 patient networks and 28 cell-line networks for following analysis. Among them, 17 common networks were extracted. They include cancer cell line or patient-specific genomic alterations and are independent of tissue origin and cancer type; instead, cancer cell lines or patients are specifically described by differentially wired networks with distinct network topology that result from the genomic alterations. The different cancer cell lines or patients are subsequently clustered on the basis of their network dynamics in response to the same pharmacological perturbations.

### Targets for drug simulation in p53 network

For the drug simulation of single node, we used 6 nodes (AKT, BCL2, CYCE, MDM2, WIP1, and ATM) that targeted drugs were developing or being tested. For the drug simulation of node combination, we used 15 combinations of these 6 nodes as drug targets. For the drug simulation of single links, we used 27 outgoing links of the above 6 nodes as drug targets. For the drug simulation of link combination, we used 297 combinations of above 27 links as drug targets except for cases in which the two links were from the same node. For the drug simulation of node-link mixed combination, we used 135 combinations as drug targets except for the cases in which the link from a node were combined with that node.

### Simulation of drug effect with different dose

The effect of drug was implemented probabilistically at a dose from 0 to 1^8^. If a dose of a drug is 1, then drug-target node or link is fully inhibited by fixing the state of the node or weight of the link as 0, respectively. If a dose of a drug is 0, then drug-target node or link functions with the original network dynamics. If a dose of a drug is x between 0 and 1, then drug-target node or link is partially inhibited during the simulation steps at the probability of x without a bias of patterning.

### Drug response simulation

From an initial state, the logic for every node was updated in 100 simulation steps to calculate the steady dynamics of the network after previous 100 simulation steps of transient dynamics. We determined that 100 steps were enough to capture transient dynamics, because all trajectories of initial states in the attractor landscape of the networks converge on their attractors within 100 simulation steps. We defined the activity of a node as the average state value in the steady dynamics. The phenotype of each initial state was categorized by a set of activities. We defined phenotype as “cell death” if the activity of CASP3 was higher than a threshold of 0.9, because a constantly increased level of caspase indicates apoptosis^41^. Among all the possible initial states of a network, we obtained the ratio of initial states that converged to the cell death phenotype. We illustrated this procedure in Supplementary Fig. 1.

We obtained the dose–response curve of a network representing the ratio of states producing the cell death phenotype by simulating a drug with a dose from 0 to 1 with an interval of 0.1. We normalized this curve by adjusting the value at dose 0 to 0. The resulting dose–response curve of a network for a drug is denoted as *f* (*x*), where x is a dose of the drug in the range of 0 to 1. The corresponding dose–response curve of the control network is denoted as *g* (*x*).

With a stochastic perturbation, the state of network transition between the attractor without the perturbation and the other attractor with the perturbation, represented as “ergodic set”^23^. Analysis of the ergodic sets constructed by the simulations of different networks may help to account for differences in potency of the different dose–response curves (Supplementary Fig. 8)

### Measures for evaluating the dose–response curve

Efficacy is the maximal effect of a drug and was calculated from a dose–response curve by ${\max }(f\left(x\right))$. To evaluate potency, we defined the IC50 of a drug response curve using linear approximation as follows:

1 $$\frac{0.5-f({x}_{1})}{f({x}_{2})-f({x}_{1})}+{x}_{1},$$

where *x*_1_ or *x*_2_ is the largest or smallest dose before or after a drug response curve crossing 0.5, respectively. If efficacy of a drug response curve is less than 0.5, we noted that IC50 of the case as 1. Toxicity in normal cells was calculated as the efficacy of a dose–response curve in the control network.

### Validation of simulation

We obtained experimental IC50 and AUC values from GDSC^26^, and compared to simulated IC50 and AUC, respectively. We obtained GR50, Grmax, and GR area over the curve (AOC) values from Grbrowser^28^, and compared to simulated IC50, efficacy, and 1-AUC, respectively. For comparison, we categorized these values to sensitive or resistant by defining thresholds. The experimental IC50 values were categorized by the threshold of 0.5 after normalizing the value of IC50 to the range of 0 to 1 using the information about the minimum and maximum concentrations of the tested drug. The experimental AUC values were categorized by the average value. The GR50 values were categorized by the threshold of 0.5. The drugs with infinite GR50 were excluded for comparison. The Grmax values were categorized by the threshold of 0.5. The GR_AOC values were categorized by the threshold of 0.5. The simulated IC50 and AUC values were categorized by the threshold of 0.5 and the simulated efficacy value was categorized by the threshold of 0.8 under the condition that the DNA damage in the cell line-specific networks was OFF (0). The predictive rate for each drug was calculated by comparing whether the simulated and experimental values were sensitive or resistant in corresponding cell lines.

We further compared our cell line-specific predictions with random predictions acquired from networks with randomized genomic alterations. We obtained dose–response curves by simulating the effects of the inhibiting the targets on 100 networks with random alterations.

To validate drug combination effects, we obtained synergy scores of drug combinations from DREAM challenge^31^. For the comparison, a combination index was calculated from the IC50 values from simulating A, B as follows:

2 $${{{{{\mathrm{CI}}}}}}=\frac{{{{{{\mathrm{IC}}}}}}{50}_{{{{{{\mathrm{A}}}}}},\;{{{{{\mathrm{B}}}}}}}}{{{{{{\mathrm{IC}}}}}}{50}_{{{{{{\mathrm{A}}}}}}}}+\frac{{{{{{\mathrm{IC}}}}}}{50}_{{{{{{\mathrm{A}}}}}},\;{{{{{\mathrm{B}}}}}}}}{{{{{{\mathrm{IC}}}}}}{50}_{{{{{{\mathrm{B}}}}}}}}$$

A CI value of less than 1 indicates a synergistic effect, 1 indicates an additive effect, and a value greater than 1 indicates an antagonistic effect.

### Drug response categorization

For drug response categorization, we defined an efficacy test as ${\max }\left(f\left(x\right)\right)$ > 0.8, a toxicity test as ${\max }\left(f\left(x\right)\right)-{\max }(g\left(x\right))$ > 0.5, and and a potency test as existence of *x* that satisfies $f\left(x\right)-g\left(x\right) \, > \, 0.5$. We clustered the simulated drug responses into selective control categories based on the efficacy test indicating the level of response by perturbing specific nodes or links in the network, and optimal control categories based on potency and toxicity tests indicating differences in responses between cancer and normal networks. The drug responses of cancer-specific networks were simulated under the condition that the DNA damage in the cell line-specific networks was ON (1). With 4 possible selective control categories and 3 possible optimal control categories, we can classify the responses to inhibition of specific targets into 12 groups (S_1_,O_1_)–(S_4_,O_3_). If a drug targeting a node satisfies the efficacy test, the category for selective control is S_1_ or S_2_, depending on if a drug targeting a link of the node satisfies the efficacy test or not. If a drug targeting a node do not satisfy the efficacy test, the category for selective control is S_3_ or S_4_, depending on if a drug targeting a link of the node satisfies the efficacy test or not. If a drug satisfies the toxicity test, the category for optimal control is O_1_. Otherwise, category for optimal control is O_2_ or O_3_, depending on if the drug satisfies the potency test or not. In total, we classified the drug response into 12 categories. We marked the 6 categories with S_1_/S_2_/S_3_ and O_1_/O_2_ as D responses, and the rest, any with S_4_ or O_3_ as U responses. The therapeutic window could be estimated as difference between simulated minimum effective dose (sMED) and simulated maximum tolerate dose (sMTD), which were calculated by doses reaching a threshold 0.25 from cancer and control networks, respectively.

### Therapeutic window for combination drugs

To identify therapeutic windows, we simulated drugs in a combination set with different doses and dose–response landscape can be obtained as *f*(*x*,*y*), where *x* and *y* are doses for two drugs. We visualized the therapeutic window by calculating $f(x,y)-g\left(x,y\right)$ as optimality.

### Identification of critical determinant and dominance relationship

We defined the critical determinant of the response of a cancer network to a drug as the minimum genomic alterations that dominantly determined the response. The critical determinants were obtained as follows: (1) generate subnetworks with all possible combinations of genomic alterations in the original cancer network; (2) obtain dose–response curves through drug simulations from all the subnetworks and the control network; (3) plot a two-dimensional efficacy–potency map representing all the dose–response curves; (4) obtain a set of networks near the original cancer network in the efficacy–potency plot, for which the distance to the original network is less than 0.1, that exhibited the same drug response curve; (5) obtain the common and minimum genomic alteration(s) in this set of networks. These minimum genomic alterations are the critical determinants. We exemplified all the possible cases of identifying the critical determinants for a network with two genomic alterations in Supplementary Fig. 4.

After obtaining all the critical determinants for cancer networks by repeating this procedure, we obtained a multi-level dominance relationship between the critical determinants as follows: (1) generate each test network for every combination containing one desirable and one undesirable critical determinants; (2) obtain dose–response curves through drug response simulations of all the test networks; (3) examine which of the two critical determinants is more dominant by determining whether the response of a test network is desirable or undesirable; (4) sort all the critical determinants so that the more dominant critical determinant is in a higher position; (5) classify the critical determinants in the lowest position as level 1 and the critical determinants in the higher position as the higher level.

### Statistics and reproducibility

A Wilcoxon rank sum test was performed to calculate the statistical significance of the difference in correlation coefficients between random predictions and cell line-specific predictions.

### Reporting summary

Further information on research design is available in the Nature Research Reporting Summary linked to this article.

## Supplementary information

Supplementary Information

Description of Additional Supplementary Files

Supplementary Data 1

Supplementary Data 2

Supplementary Data 3

Supplementary Data 4

Supplementary Data 5

Supplementary Data 6

Supplementary Data 7

Supplementary Data 8

Supplementary Data 9

Reporting Summary

## Data Availability

All data generated or analyzed during this study are included in this published article (and its supplementary files).

## Code availability

The simulation codes written in Matlab and Boolean network datasets are provided in the Zenodo repository 10.5281/zenodo.6979237^42^.
