# Supplementary material for: Evaluating a therapeutic window for precision medicine by integrating genomic profiles and p53 network dynamics
Source: Commun Biol. 2022 Sep 7;5:924. doi: 10.1038/s42003-022-03872-1 (PMC9452682; doi:10.1038/s42003-022-03872-1)
Supplement: Supplementary file 1 — Supplementary Information [file 42003_2022_3872_MOESM1_ESM.pdf]

Evaluating a therapeutic window for precision medicine by integrating genomic profiles and p53 network dynamics

Minsoo Choi[1†], Sang-Min Park[1,2†], Kwang-Hyun Cho[1*]

[1]Department of Bio and Brain Engineering, Korea Advanced Institute of Science and Technology (KAIST), Daejeon 34141, Republic of Korea

[2]College of Pharmacy, Chungnam National University, Daejeon, 34134, Korea

This file includes:

Supplementary Note

Supplementary Figures 1 to 8

Other supplementary materials for this manuscript include the following:

Supplementary Data 1 to 8

[†]These authors contributed equally.

*Corresponding author. E-mail: ckh@kaist.ac.kr, Phone: +82-42-350-4325, Fax: +82-42-350-4310, Web: http://sbie.kaist.ac.kr/

Supplementary Note

Validation of toxicity with clinical and pre-clinical data

Phase I clinical trials are the first step to administrate drugs on humans. The main purpose of phase I clinical trials is to identify the toxicity level of a new drug by measuring Maximum tolerated dose (MTD). For comparison, we calculated simulated MTD (sMTD) in our simulation framework such that it can be translational to clinical applications (Supplementary Text Fig. 1) and validate its usefulness by using both pre-clinical and clinical data.

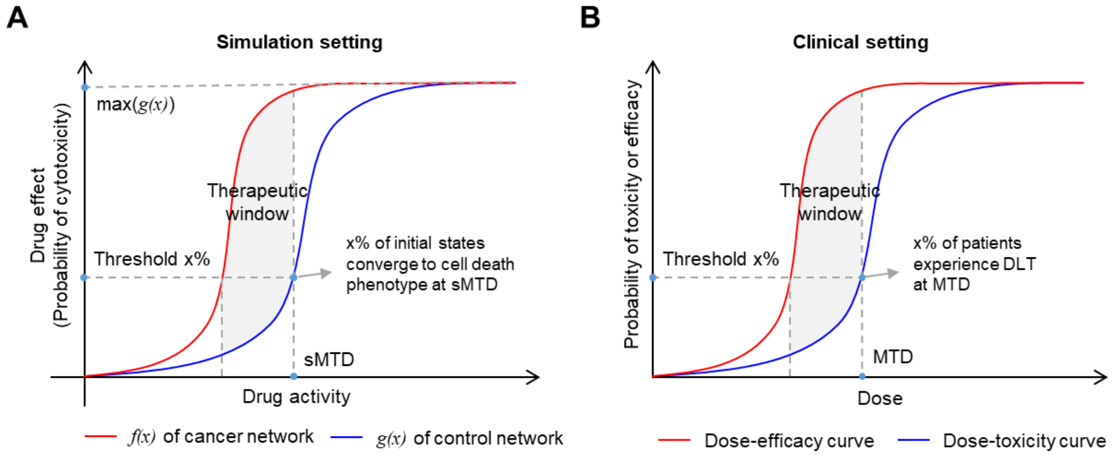

Supplementary Note Figure 1. Comparison of therapeutic windows evaluated in our simulation framework or clinical trials.

The traditional dose-escalation methods for phase I clinical trials increase the dose of a drug up to MTD where a certain percentage of patients (typically 20% to 33%) experience dose-limiting toxicity (DLT), a clinically severe adverse event (Supplementary Text Fig. 1B). We note that this process is similar to our simulation framework of obtaining a dose response curve (Supplementary Text Fig. 1A). Here, we assumed that cancer and control networks can represent tumor and normal tissues of a cancer patient, respectively. Heterogeneous patients in a clinical trial can be represented by different initial states of networks. The dose response curve of the control network was calculated as percentages of initial states according to different levels of drug activity that converge to the cell

death phenotype, representing DLT. From this perspective, we calculated sMTD of a drug where 25% of initial states converge to the cell death phenotype in the control network.

We collected clinical trials conducted with the drugs used for in vitro experimental validation in Fig. 2. Among seven drugs, only data for three drugs are available in Phase I clinical trials to obtain MTD: two AKT inhibitors, dactolisib and omipalisib, and one BCL2 inhibitor, navitoclax (Supplementary Text Table 1).

Supplementary Note Table 1. MTDs of drugs measured in clinical studies.

**Drug**	**Target**	**Cancer type**	**MTD** (per day)	**ClinicalTrials.gov Identifier**	**PMID**
Dactolisib (BEZ235, NVP-BEZ235)	AKT	Advanced Solid Malignancies Enriched by Patients With Advanced Breast Cancer	1000 mg (capsule) 1200 mg (sachet)	NCT00620594	29882016
		Advanced Renal Cell Carcinoma	Poorly tolerated	NCT01453595	27286790
		Transitional Cell Carcinoma	Poorly tolerated	NCT01856101	26779597
		Advanced Pancreatic Neuroendocrine Tumors	Poorly tolerated	NCT01658436	26851029
Omipalisib (GSK458, GSK2126458)	AKT	Advanced Solid Tumor Malignancies	2.5 mg	NCT00972686	26603258
Navitoclax (ABT-263)	BCL2	Small Cell Lung Cancer (SCLC) or Other Non-Hematological Malignancies	250 mg	NCT00445198	21282543
		Relapsed or Refractory Lymphoid Malignancies	325 mg	NCT00406809	21094089

In case of dactolisib, its MTD is at most 1200 mg/day among breast cancer patients while it depends on the drug formulation. However, dactolisib is poorly tolerable with high incidence of DLTs in patients with other cancer types, which results in termination of trials without measuring

MTD. In case of omipalisib, MTD is 2.5 mg/day, whereas for navitoclax of which MTD is dependent on cancer types, has the highest MTD at 325 mg/day. In our simulation, MTD for inhibition of AKT and BCL2 are 0.46 and 0.85, respectively, which indicates that BCL2 inhibition is more tolerable than that of AKT inhibition. Consistently, MTD of navitoclax is higher than that of omipalisib except dactosilib with mixed results.

Toxicity measured in animal study can contribute to prediction for toxicity in clinical study. Because the number of comparable clinical data is limited, we also performed a comparison with pre-clinical data using mouse models. Among the in vivo studies conducted with drugs in Fig. 2, we collected the measured MTD or the largest dosage per drug used in the previous studies (Supplementary Text Table 2).

Supplementary Note Table 2. Drug doses used in pre-clinical studies (PO: per oral, SC: subcutaneous, IV: intravenous, IP: intraperitoneal).

Drug	Target	Administration	Dose	PMID
Dactolisib	AKT	PO	45 mg/kg	21966435
Omipalisib	AKT	PO	3 mg/kg	28537878
Navitoclax	BCL2	PO	100 mg/kg	18085673
Nutlin-3	MDM2-p53	PO	200 mg/kg	14704432
Purvalanol A	CYCE	IV	30 mg/kg	16914540
Nutlin-3	MDM2-p53	IV	20 mg/kg	20947617
Purvalanol A	CYCE	IP	20 mg/kg	23341542
RO-3306	CYCE	IP	4 mg/kg	27385216
KU-55933	ATM	IP	10 mg/kg	25881002
Nutlin-3	MDM2-p53	IP	50 mg/kg	28341911
KU-55933	ATM	SC	30 mg/kg	26299580

Since routes of drug administration affect MTD such that the oral dose is usually higher than other routes, we compared the sMTD with the experimental dose by separating the drugs according to the routes of administration. The result showed a positive correlation between the

sMTD and experimental dose of several drugs (PO: r = 0.9394, IP: r =0.8310), which indicates that we can expect higher MTD in vivo with higher sMTD in simulation (Supplementary Text Fig. 2). As the actual toxicity measurement in Phase I clinical trials determines usage based on the results obtained from animal testing, our results can provide important information for estimating toxicity in actual clinical tests.

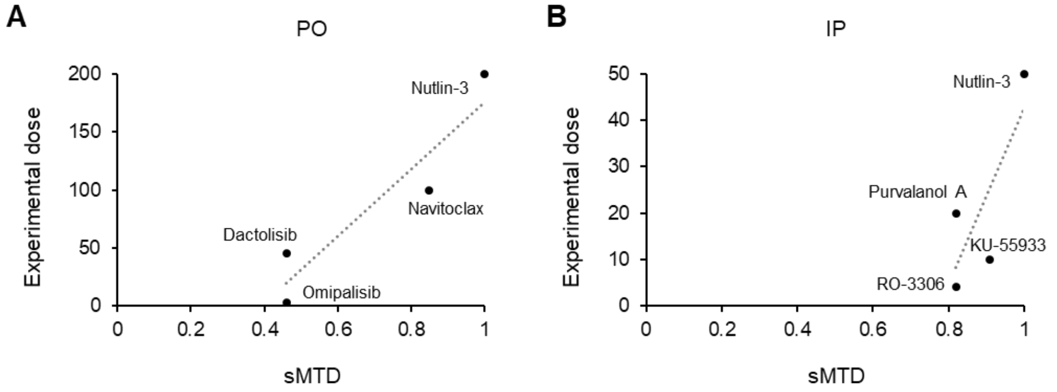

Supplementary Note Figure 2. Comparison of sMTD and in vivo drug toxicity administrated (A) per oral or (B) intraperitoneal.

Design of optimal combination therapeutic strategies for improving therapeutic window

Our framework identified three different strategies for enhancing the therapeutic window: improving efficacy of the cancer network response, increasing potency of the cancer network response than the control network response, or reducing toxicity of the control network response. As an example, we showed in NT_8 that inhibiting MDM2-p53 and WIP1 produced a desirable response with efficacy, whereas individually their inhibition had no efficacy (Supplementary Fig. S6A). We could have used the critical determinant and dominance analysis to predict the effectiveness of MDM2-p53 and WIP1 combined inhibition. In the critical determinants of single p53-MDM2 inhibition (Supplementary Fig. 5B), NT_8 had activated CYCG and activated WIP1 alterations, which generated an undesirable response (Supplementary Data 6). Inhibiting the molecules in this critical determinant of undesirable response is an intuitive option for drug

combination. Indeed, simulations of the combinatorial inhibition of p53-MDM2 and WIP1, which targets the critical determinant for inhibition of p53-MDM2, was effective in achieving a therapeutic window in NT_8, which has activated CYCG and inactivated ARF alterations that are the critical determinants of the response to this combination (Supplementary Data 6).

As another example, we identified combinations with increased potency in cancer networks than the control network. The combination of AKT and WIP1 inhibition produced a desirable response (O_2) with a therapeutic window, whereas individually AKT inhibition produced no greater potency (O_3) in cancer networks of NT_9, 19, and 24 than in the control network (Supplementary Fig. S6B). We found that these networks showed an undesirable response to WIP1 inhibition by the critical determinant of activated AKT (Supplementary Data 6). In addition to WIP inhibition, the combination with AKT inhibition to block the effect of this critical determinant enhanced the therapeutic window producing a desirable response in theses cancer networks.

We also identified combinations with reduced toxicity in the control network. AKT inhibition was toxic in the control network and many cancer networks showed insufficient difference in potency between the cancer and control network. Combination inhibition of AKT and ATM reduced the toxicity at the control network producing a O_1 response compared with the O_3 response of AKT inhibition alone in NT_6, 9, 13, and 15 (Supplementary Fig. S6C, top). The networks had an activating WIP1 alteration or inhibiting MDMX and BAX alterations as the critical determinants (Supplementary Data 6). In NT_8, 17, 29, and 34, AKT and ATM inhibition also enhanced the therapeutic window resulting in an O_1 response, whereas AKT inhibition alone produced an O_2 response (Supplementary Fig. S6C, middle). These networks had critical determinants of either activated CYCG or activated MDM2 alteration (Supplementary Data 6), suggesting that other networks with these critical determinants would exhibit a higher therapeutic window by the combination of AKT and ATM inhibition compared with the therapeutic window of AKT inhibition alone. Note that only are the critical determinants of the response to combined inhibition different between these groups of networks, but their dose response curves differ, yet both

groups of networks achieve a better response through the combination. Another combinatorial target for reducing the toxicity of AKT inhibition was MDM2-E2F1 inhibition. This combination produced a desirable response in the largest set of networks among all the 480 drug combinations (NT_3, 5, 9, 11, 16, 19, 24, 25, 30, and 33) (Supplementary Fig. S6C, bottom).

Drug synergism analysis

To estimate the synergism in drug combinations, we measured CI for the 447 drug combinations that we simulated for all cancer networks. As a result, the effect of most drug combinations was additive and only 10% of drug combinations showed synergistic effects (Supplementary Fig. 7a). Among the synergistic combinations, we found that those with link inhibition were enriched over those that were only a combination of node inhibition (Supplementary Fig. 7b). This suggested that selective inhibition of specific connections between molecules in a network may provide a path for further therapeutic improvement.

Functional feedback analysis

The p53 regulatory network contains 160 negative and 228 positive feedbacks (Supplement Data 7). Since cancer has mutations that are continuously activated or deactivated, the value of the mutant node in the cancer network was fixed and thus that node could not function as feedback. Excluding feedback whose influence is limited by the genomic profile of cancer networks allows us to identify functional feedbacks responsible for the phenotype. Consequently, each cancer network had a different set of functional feedbacks.

Here, we highlighted the functional feedbacks related with the simulated drug responses of NT_8 and NT_9 in Figure 4. Both NT_8 and NT_9 showed high efficacy by high-dose of AKT perturbation, but only NT_9 showed high efficacy sensitively to low-dose. Comparing the functional feedbacks between the two NTs, there were 25 differences. The longer the feedback circuit, the smaller the effect[1, 2], so we defined the shortest as critical functional feedback. The

critical functional feedbacks for NT_9, which NT_8 does not have, were negative feedbacks for p53 of length three as follows:

p53 —> Cyclin G —┤ ATM —> p53

p53 —> Cyclin G —> Mdm2 —┤ p53

Because NT_9 more adequately regulates p53 activity under the influence of these negative feedbacks, it can inhibit p53-induced apoptosis when given AKT perturbation, making this network relatively less sensitive than NT_8. On the other hand, NT_8 is sensitive to AKT perturbation because this feedback group is not functional due to the CYCG mutation, which is a critical determinant (CD) for AKT perturbation. In this study, we demonstrated that CD and its relationship determine different drug responses. This result suggests that CDs might be an important node for critical functional feedback. The association between functional feedback and CD, which are key components of drug response, will be further investigated in subsequent studies.

Supplementary Figures

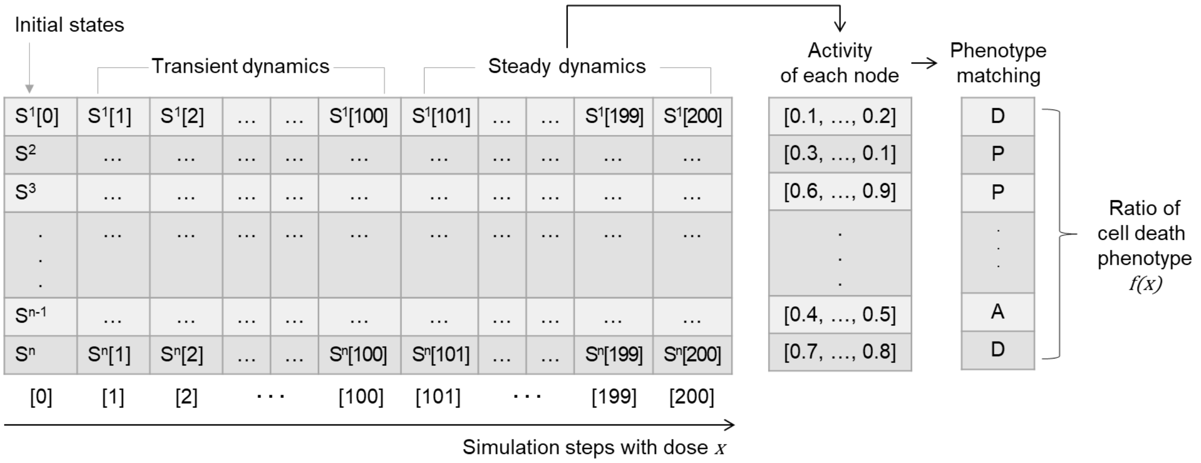

Supplementary Figure 1. Procedure to obtain the dose response curve. Given a Boolean model for a network has n nodes, we perform Boolean simulations from all the initial states by perturbing the drug target node with dose x, calculate the activity of each node from the steady dynamics in simulation results, match phenotypes to each state, and finally calculate the ratio of cell death phenotype. Repeat this procedure with different doses in the range of 0 to 1 to obtain the dose response curve *f(x)*.

a

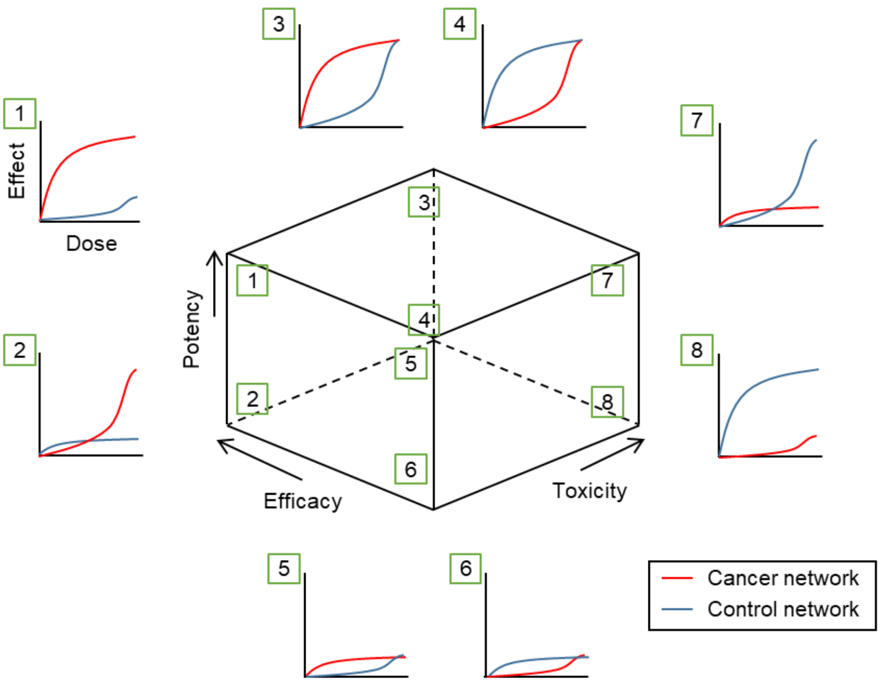

b

Pattern	Efficacy test (node)	Toxicity test	Potency test	Selective control category	Optimal control category
1	Y	Y	Y	S_{1-2}	O_1
2	Y	Y	N	S_{1-2}	O_1
3	Y	N	Y	S_{1-2}	O_2
4	Y	N	N	S_{1-2}	O_3
5	N	N	N	S_{3-4}	O_3
6	N	N	N	S_{3-4}	O_3
7	N	N	N	S_{3-4}	O_3
8	N	N	N	S_{3-4}	O_3

Supplementary Figure 2. Possible patterns of the dose response curve. (a) From the dose dependent simulations of the different p53 networks with different drug perturbations, the resulting efficacy, potency, and toxicity values of each dose response curve were plotted in the 3-dimensional space. The position within the space for each pattern of response is indicated by numbers. **(b)** Drug response categorization for the eight patterns in (a) based on selective and optimal control categories using the efficacy, toxicity, and potency tests in Fig. 3a. These examples are comparable to the simulation results in Fig. 3b. Here, the above patterns were assumed to be the result of dose-dependent perturbations for a node, and the evaluation of dose-dependent perturbations for the node's outgoing links can finally determine the selective control category.

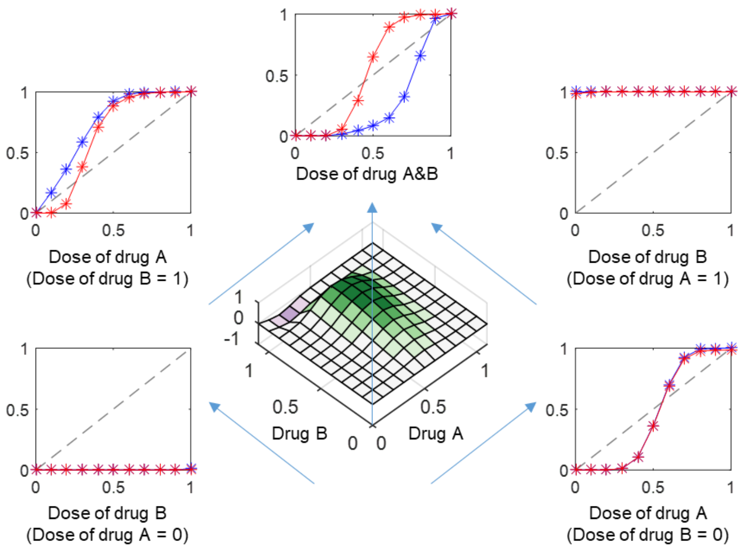

Supplementary Figure 3. Interpretation of the dose response landscape for combination. After dose dependent simulations of drug combinations, we obtained a dose response landscape representing the difference of drug effects between cancer and normal networks. This landscape can be analyzed in the simplified form of a dose response curve by simulating the same dose for both drugs in the combination, which is the topmost graph obtained from the information on the diagonal of the dose response landscape.

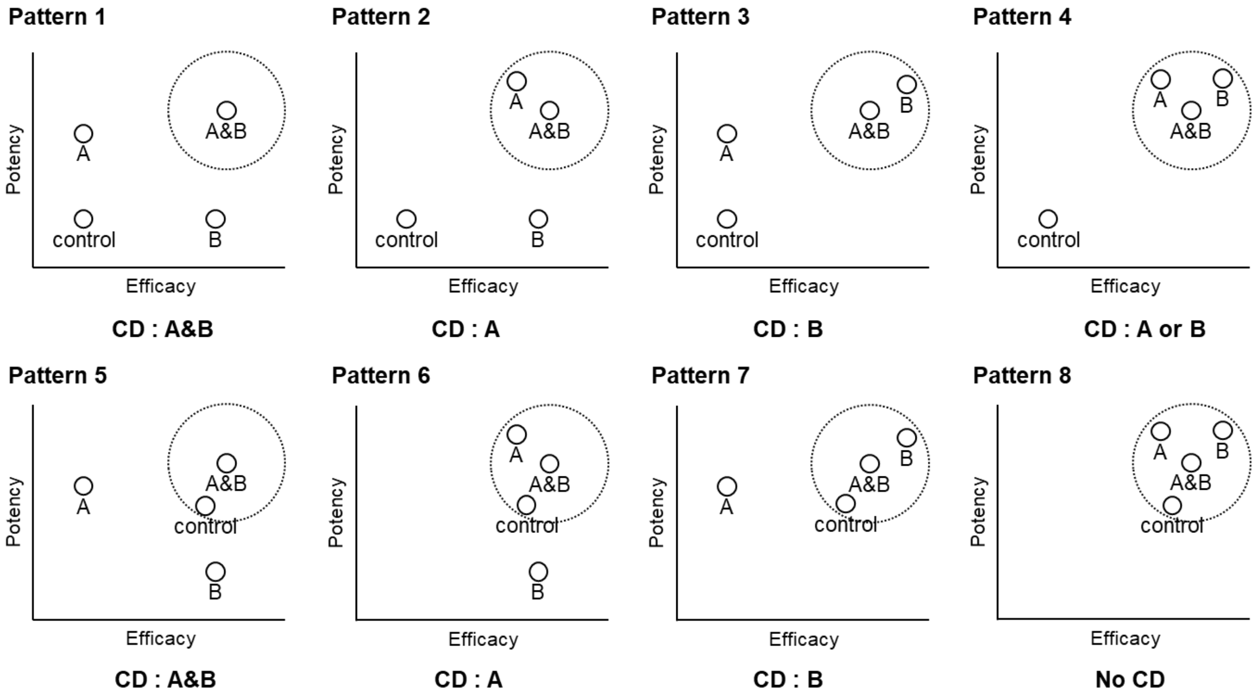

Supplementary Figure 4. Critical determinants for networks exhibiting different drug responses. All possible cases of critical determinants in networks with two genetic alterations, A and B. CD; critical determinants.

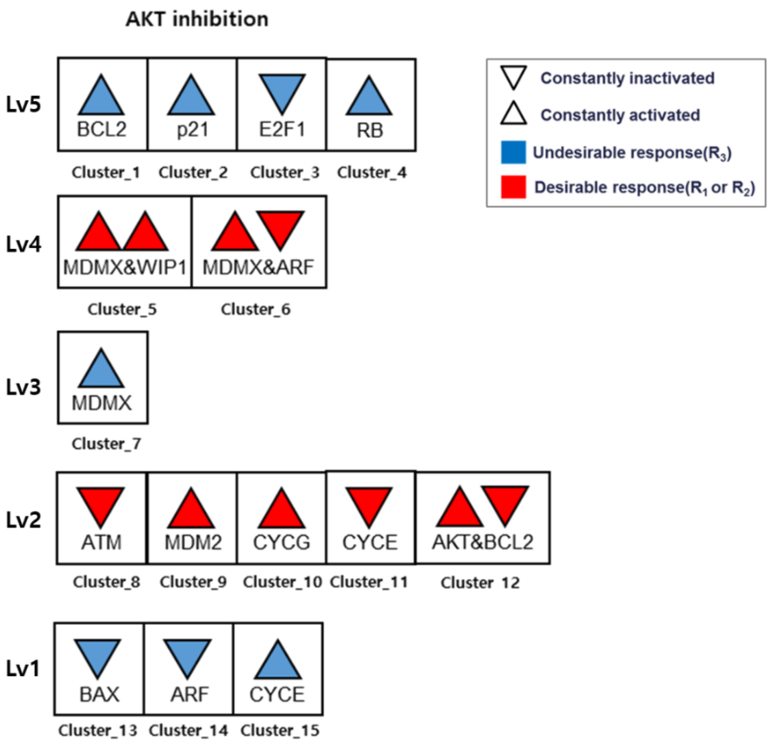

Supplementary Figure 5. Critical determinants and their dominance relationship for AKT inhibition using the patient-specific networks. Upward pointing triangle indicates an activating genetic alteration; downward pointing indicates inactivating genetic alteration. Red indicates a D response; blue indicates an U response. The more dominant critical determinants in higher positions increase the level number (Lv).

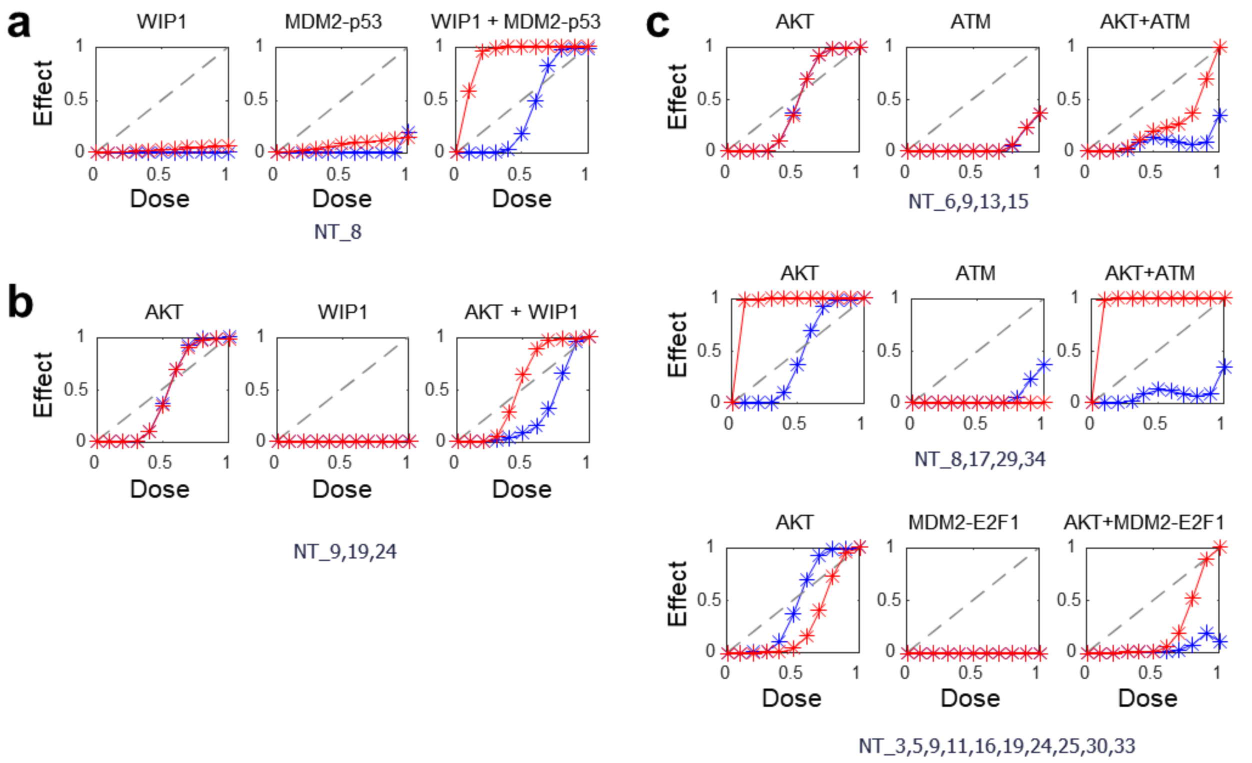

Supplementary Figure 6. Three strategies of drug combinations for enhancing therapeutic windows. The cases of enhanced therapeutic windows by increasing the efficacy **(a)**, the potency **(b)**, or reducing the toxicity **(c)**.

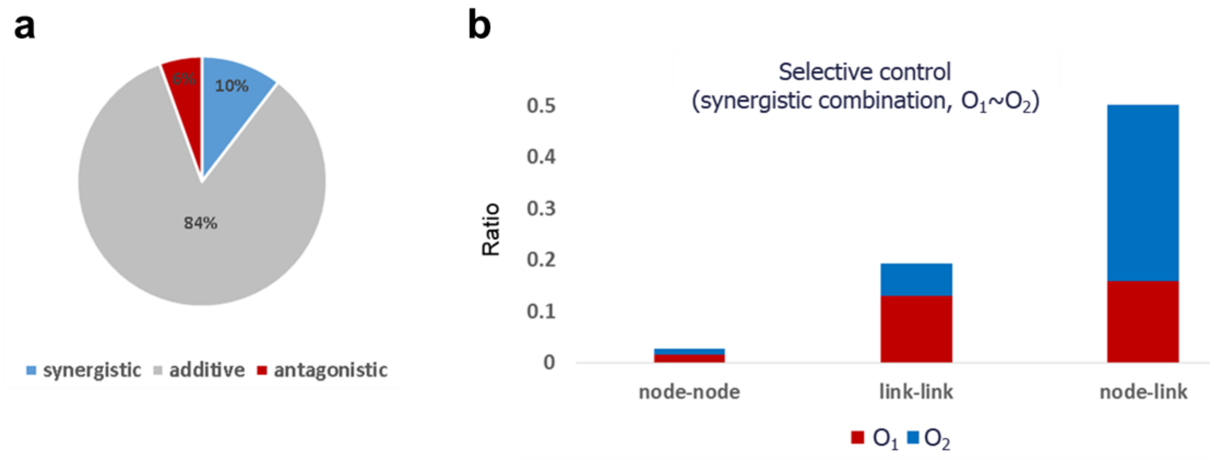

Supplementary Figure 7. Effects of combination perturbations. (a) The ratios of synergistic, additive, or antagonistic effects among 447 combination perturbations in cancer cell-specific networks. **(b)** The ratios of node-node, node-link, or link-link perturbations among synergistic combinations showing O_1 or O_2.

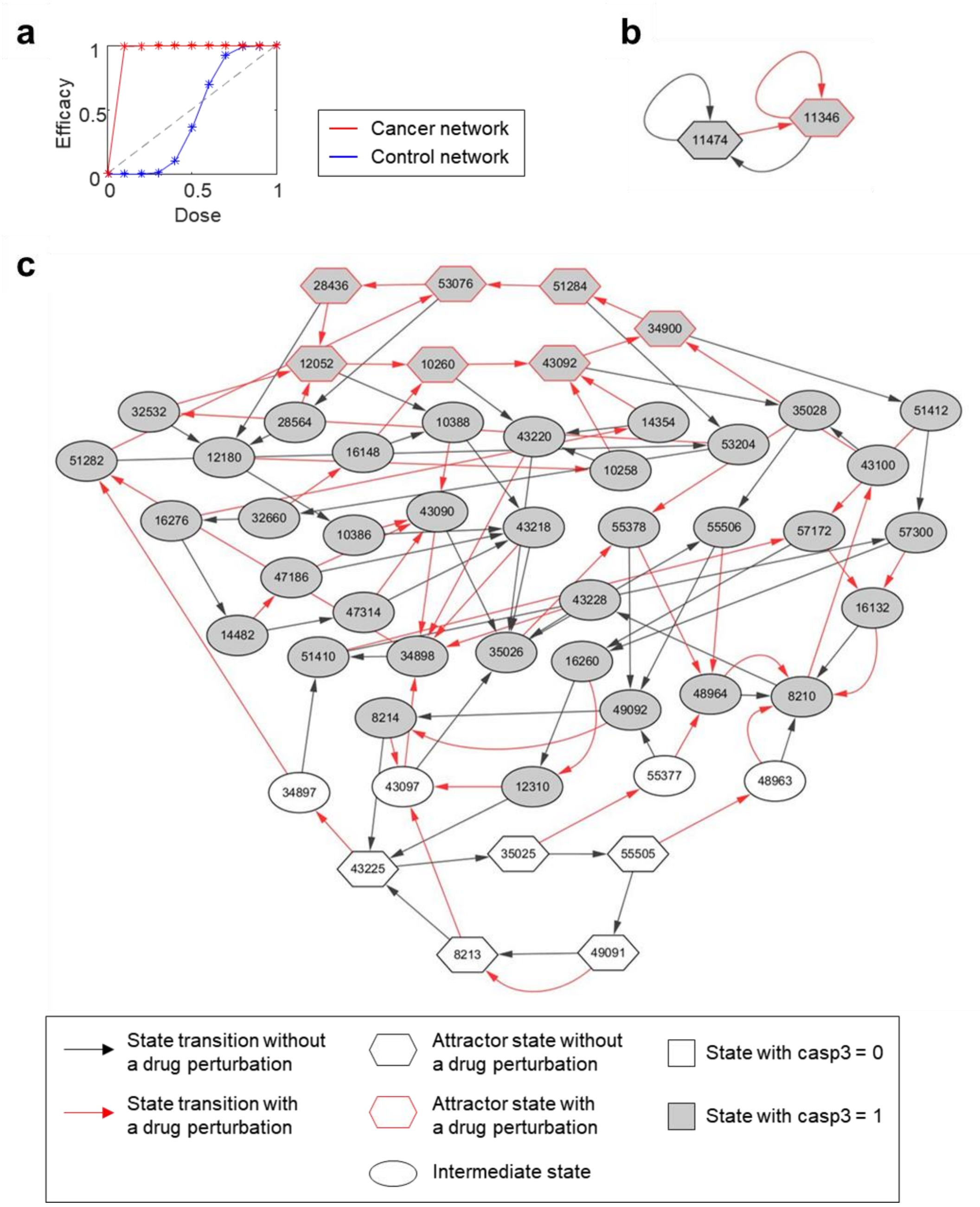

Supplementary Figure 8. Analysis of ergodic set. (a) Dose response curves of NT8 and the control network with AKT inhibition. The ergodic sets of **(b)** NT8 and **(c)** the control network from the simulations with AKT inhibition. The number represents the value of a network state converted to decimal. The original attractor landscape of NT8 without a drug perturbation had the point attractor (denoted as 11474) that corresponds to cell death phenotype (casp3 = 1) with relatively small basin. However, other attractors in the original attractor landscape were not involved in the ergodic set of NT8 by AKT inhibition. The attractor state after AKT inhibition (denoted as 11346) corresponded

to cell death phenotype. Thus, NT8 showed a very potent dose response curve that a small dose of AKT inhibition was enough to fully induce cell death phenotypes for the network. Contrary to NT8, the ergodic set of the control network by AKT inhibition included a cyclic attractor (denoted as 43225, 35025, 55505, 59091, and 8213), which has the largest basin in the original attractor landscape of the control network and does not correspond to the cell death phenotype (bottom area). On the other hand, the attractor state after AKT inhibition (denoted as 28436, 12052, 10260, 43092, 34900, 51284, and 53076) corresponded to cell death phenotype (top area). As the dose of a drug for AKT inhibition increase, the network will more frequently transits from non-cell death to cell death states during the simulation steps. Thereby, the dose response curve of the control network increased with the dose for AKT inhibition.
