# Supplementary material for: Evaluating a therapeutic window for precision medicine by integrating genomic profiles and p53 network dynamics
Source: Commun Biol. 2022 Sep 7;5:924. doi: 10.1038/s42003-022-03872-1 (PMC9452682; doi:10.1038/s42003-022-03872-1)
Supplement: Supplementary file 2 — Description of Additional Supplementary Files [file 42003_2022_3872_MOESM2_ESM.pdf]

Description of Additional Supplementary Files

File name: Supplementary Data 1

Description: Mapping of genomic profiles (The source data of Figure 1)

File name: Supplementary Data 2

Description: Validation of dose dependent simulation (The source data of Figure 2)

File name: Supplementary Data 3

Description: Drug response of cancer specific networks (The source data of Figure 3)

File name: Supplementary Data 4

Description: Triangle map of cancer specific networks (The source data of Figure 4)

File name: Supplementary Data 5

Description: Comparison of critical determinant dominant analysis and single genomic biomarker analysis (The source data of Figure 5)

File name: Supplementary Data 6

Description: Stratification of patient-specific networks to the response of AKT inhibition (The source data of Figure 5)

File name: Supplementary Data 7

Description: Critical determinants for therapeutic improving strategies

File name: Supplementary Data 8

Description: List of all feedback circuits in the p53 network

File name: Supplementary Data 9

Description: Truth tables of the p53 network
