# Supplementary material for: Evaluating a therapeutic window for precision medicine by integrating genomic profiles and p53 network dynamics
Source: Commun Biol. 2022 Sep 7;5:924. doi: 10.1038/s42003-022-03872-1 (PMC9452682; doi:10.1038/s42003-022-03872-1)
Supplement: Supplementary file 6 — Supplementary Data 4 [file 42003_2022_3872_MOESM6_ESM.pdf]

1

NT5

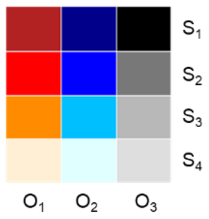
S_1
S_2
S_3
S_4
O_1
O_2
O_3

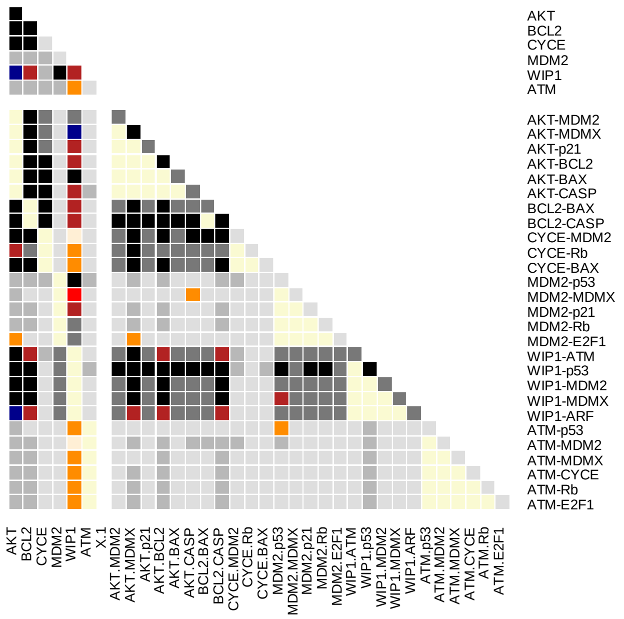
AKT
BCL2
CYCE
MDM2
WIP1
ATM
AKT-MDM2
AKT-MDMX
AKT-p21
AKT-BCL2
AKT-BAX
AKT-CASP
BCL2-BAX
BCL2-CASP
CYCE-MDM2
CYCE-Rb
CYCE-BAX
MDM2-p53
MDM2-MDMX
MDM2-p21
MDM2-Rb
MDM2-E2F1
WIP1-ATM
WIP1-p53
WIP1-MDM2
WIP1-MDMX
WIP1-ARF
ATM-p53
ATM-MDM2
ATM-MDMX
ATM-CYCE
ATM-Rb
ATM-E2F1
AKT
BCL2
CYCE
MDM2
WIP1
ATM
X.1
AKT.MDM2
AKT.MDMX
AKT.p21
AKT.BCL2
AKT.BAX
AKT.CASP
BCL2.BAX
BCL2.CASP
CYCE.MDM2
CYCE.Rb
CYCE.BAX
MDM2.p53
MDM2.MDMX
MDM2.p21
MDM2.Rb
MDM2.E2F1
WIP1.ATM
WIP1.p53
WIP1.MDM2
WIP1.MDMX
WIP1.ARF
ATM.p53
ATM.MDM2
ATM.MDMX
ATM.CYCE
ATM.Rb
ATM.E2F1

2

NT17/8/23
/2/22/28

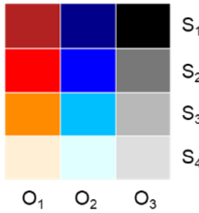

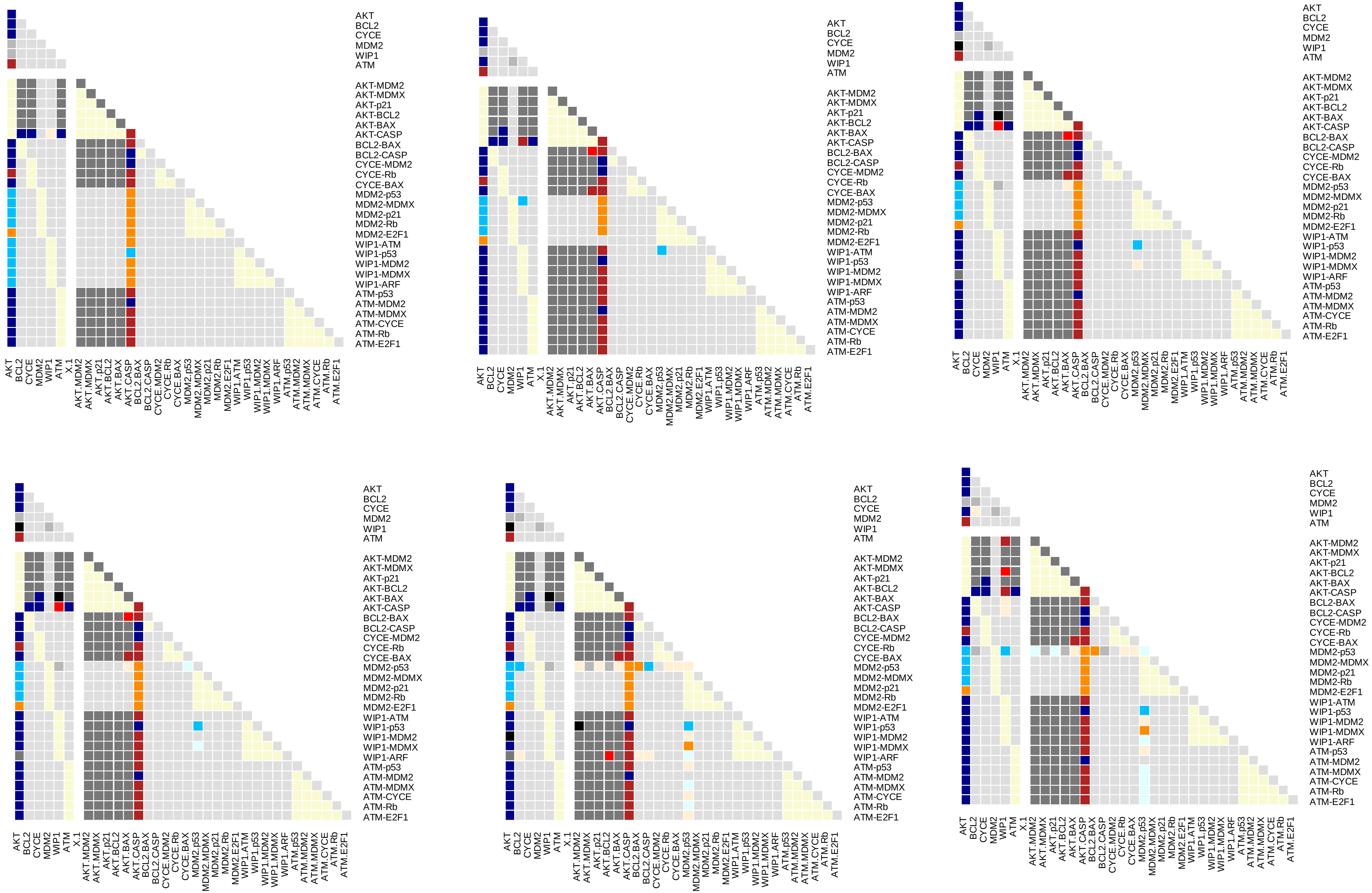

2

NT29/34/15

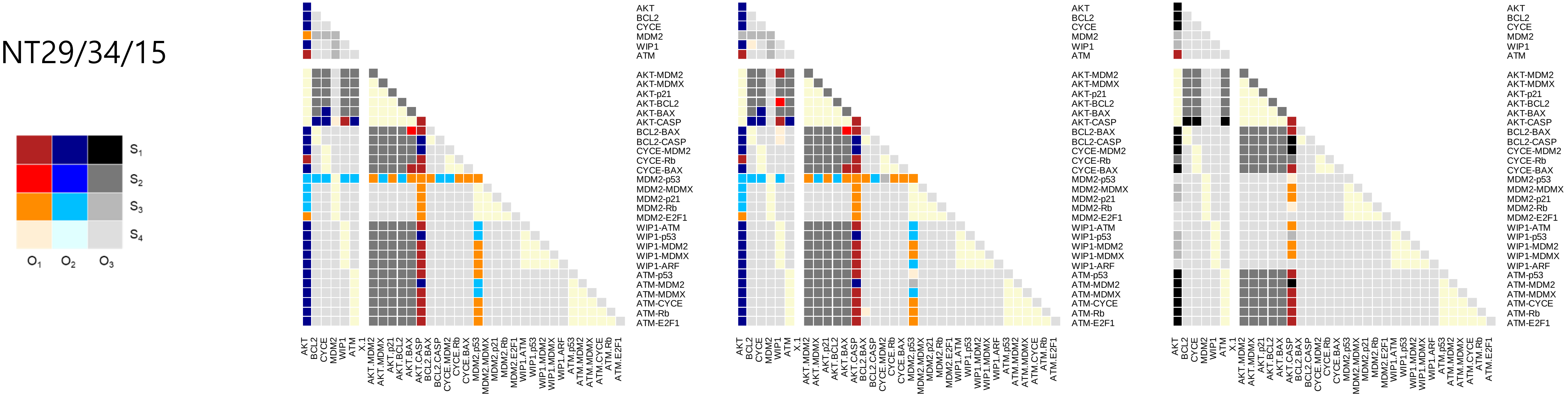
S1
S2
S3
S4
O1
O2
O3
AKT
BCL2
CYCE
MDM2
WIP1
ATM
AKT-MDM2
AKT-MDMX
AKT-p21
AKT-BCL2
AKT-BAX
AKT-CASP
BCL2-BAX
BCL2-CASP
CYCE-MDM2
CYCE-Rb
CYCE-BAX
MDM2-p53
MDM2-MDMX
MDM2-p21
MDM2-Rb
MDM2-E2F1
WIP1-ATM
WIP1-p53
WIP1-MDM2
WIP1-MDMX
WIP1-ARF
ATM-p53
ATM-MDM2
ATM-MDMX
ATM-CYCE
ATM-Rb
ATM-E2F1
AKT
BCL2
CYCE
MDM2
WIP1
ATM
X.1
AKT.MDM2
AKT.MDMX
AKT.p21
AKT.BCL2
AKT.BAX
AKT.CASP
BCL2.BAX
BCL2.CASP
CYCE.MDM2
CYCE.Rb
CYCE.BAX
MDM2.p53
MDM2.MDMX
MDM2.p21
MDM2.Rb
MDM2.E2F1
WIP1.ATM
WIP1.p53
WIP1.MDM2
WIP1.MDMX
WIP1.ARF
ATM.p53
ATM.MDM2
ATM.MDMX
ATM.CYCE
ATM.Rb
ATM.E2F1

3

NT19/24/
6/13/9

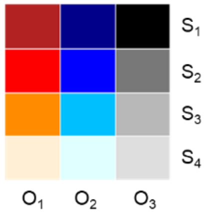

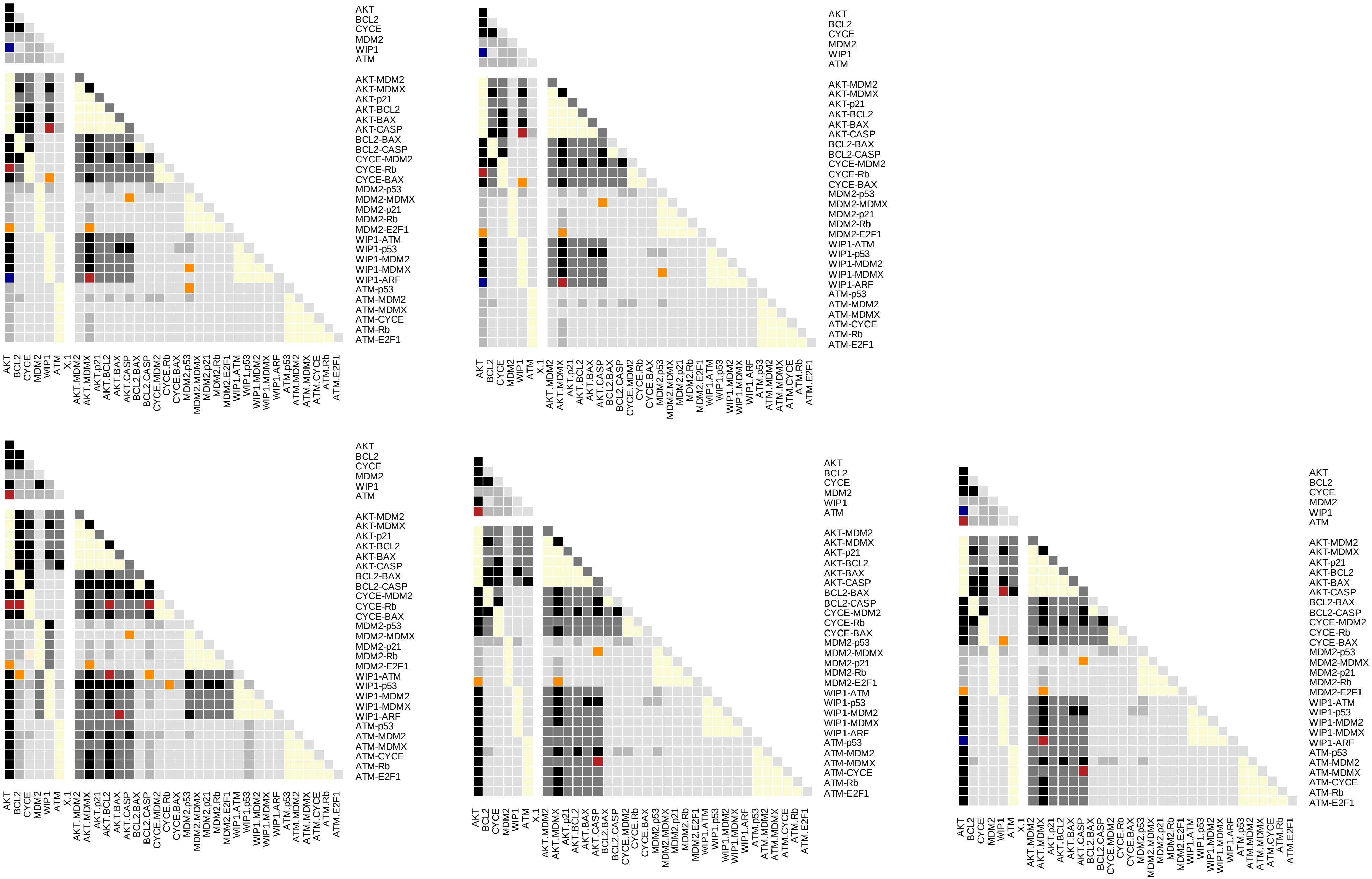

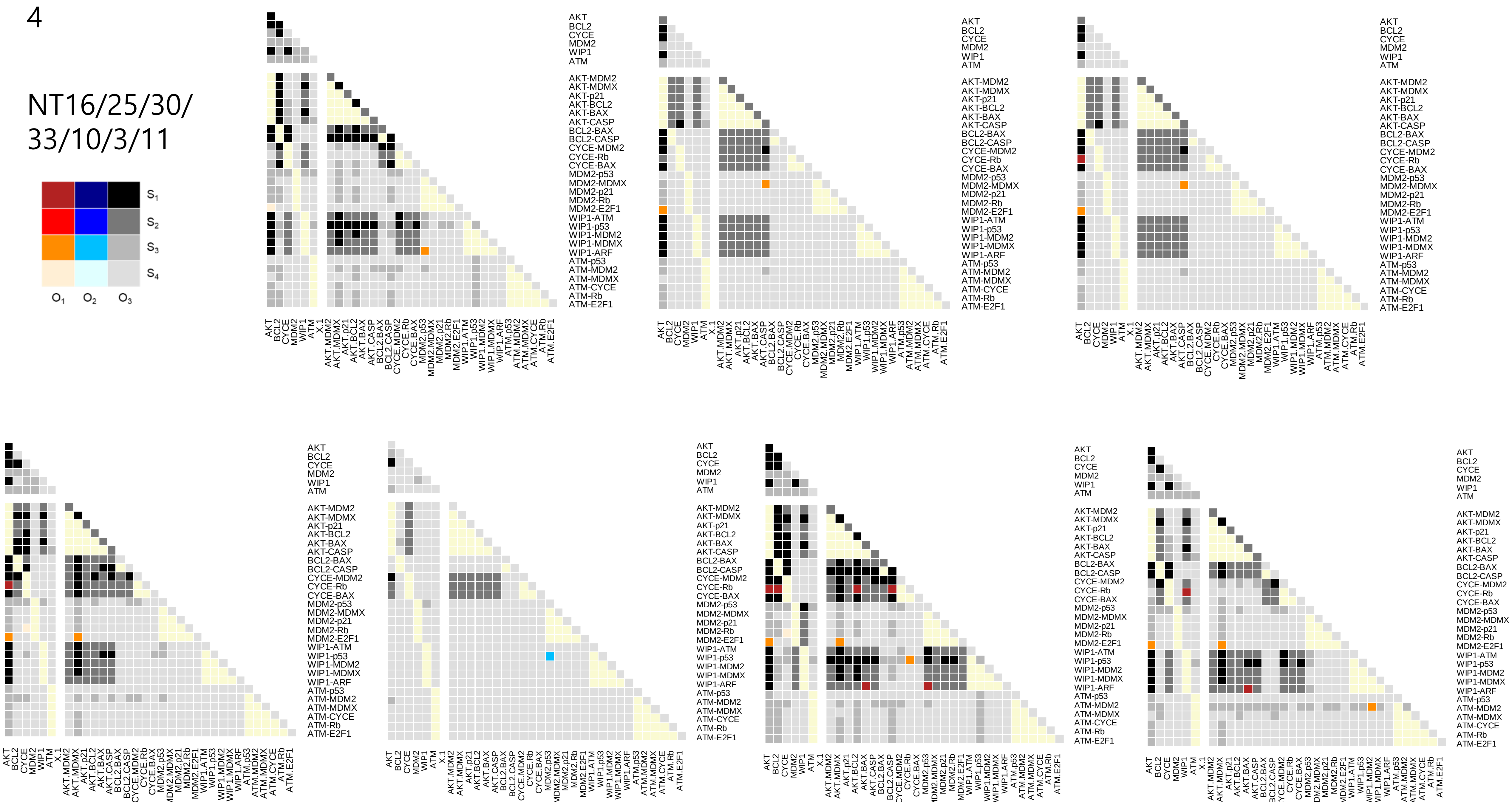

4
NT16/25/30/
33/10/3/11
S1
S2
S3
S4
O1
O2
O3
AKT
BCL2
CYCE
MDM2
WIP1
ATM
AKT-MDM2
AKT-MDMX
AKT-p21
AKT-BCL2
AKT-BAX
AKT-CASP
BCL2-BAX
BCL2-CASP
CYCE-MDM2
CYCE-Rb
CYCE-BAX
MDM2-p53
MDM2-MDMX
MDM2-p21
MDM2-Rb
MDM2-E2F1
WIP1-ATM
WIP1-p53
WIP1-MDM2
WIP1-MDMX
WIP1-ARF
ATM-p53
ATM-MDM2
ATM-MDMX
ATM-CYCE
ATM-Rb
ATM-E2F1
X.1

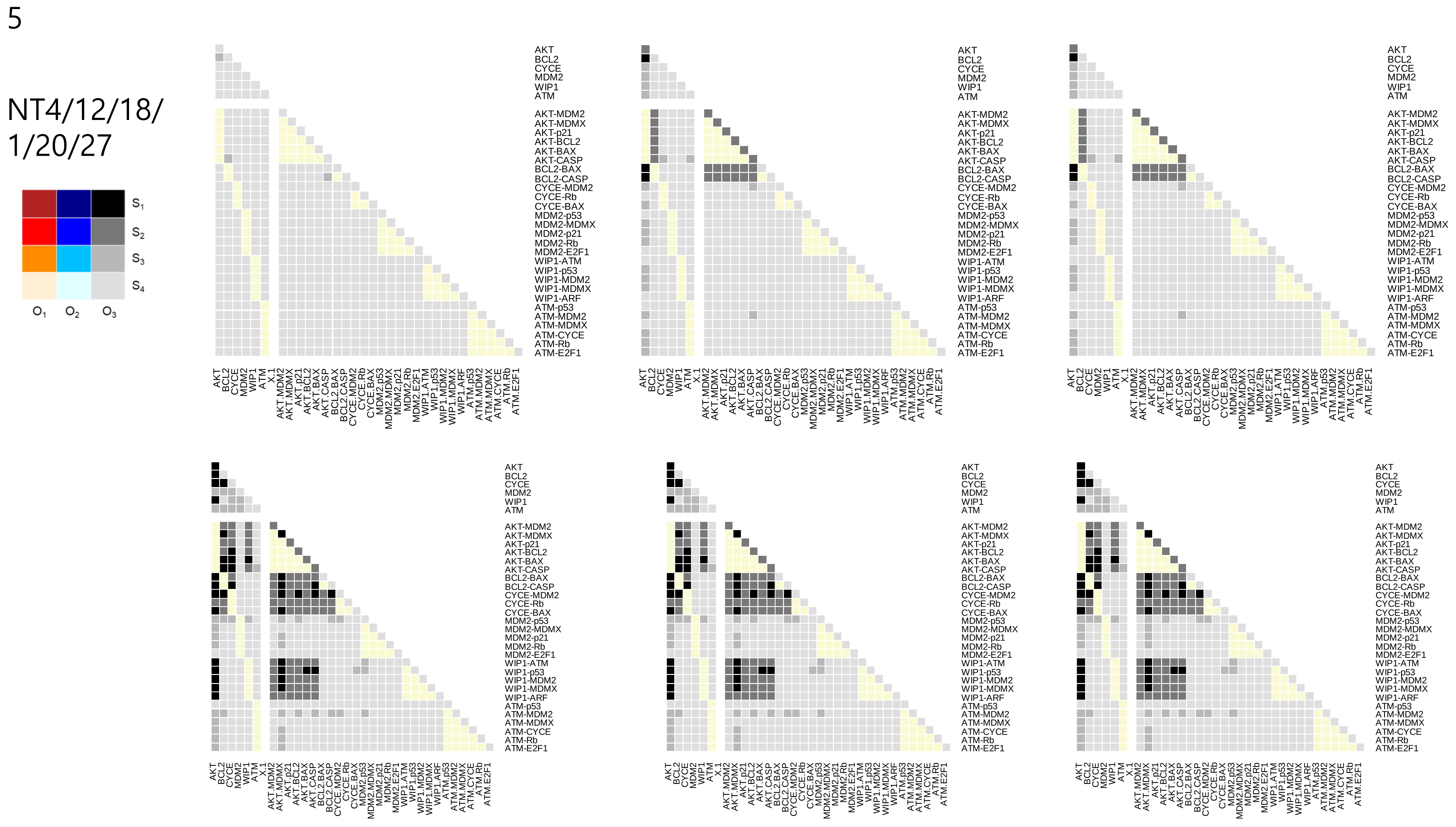

5
NT4/12/18/
1/20/27
S1
S2
S3
S4
O1
O2
O3
AKT
BCL2
CYCE
MDM2
WIP1
ATM
AKT-MDM2
AKT-MDMX
AKT-p21
AKT-BCL2
AKT-BAX
AKT-CASP
BCL2-BAX
BCL2-CASP
CYCE-MDM2
CYCE-Rb
CYCE-BAX
MDM2-p53
MDM2-MDMX
MDM2-p21
MDM2-Rb
MDM2-E2F1
WIP1-ATM
WIP1-p53
WIP1-MDM2
WIP1-MDMX
WIP1-ARF
ATM-p53
ATM-MDM2
ATM-MDMX
ATM-CYCE
ATM-Rb
ATM-E2F1
X.1
